# The anterior perforated substance (APS) revisited: Commented anatomical and imagenological views

**DOI:** 10.1002/brb3.3029

**Published:** 2023-11-27

**Authors:** Horacio José Fontana, Juan Mazzucco, Sebastián Lescano

**Affiliations:** ^1^ Hospital Central de San Isidro, Service of Neurosurgery Buenos Aires Argentina; ^2^ Instituto ARGUS de Diagnóstico por Imágenes Buenos Aires Argentina; ^3^ ARGUS Diagnóstico por Imágenes CNS imagenologist Buenos Aires Argentina

**Keywords:** anterior perforated substance, basal ganglion of Meynert, diagonal band of Broca, inferior thalamic peduncle, MRI anatomy of substantia innominata, substantia innominata, ventral amygdalofugal way

## Abstract

**Introduction:**

Since 2002, when we published our article about the anterior perforated substance (APS), the knowledge about the region has grown enormously.

**Objective:**

To make a better description of the anatomy of the zone with new dissection material added to the previous, to sustain the anatomical analysis of the MRI employing the SPACE sequence, interacting with our imagenology colleagues. Especially, we aim to identify and topographically localize by MRI the principal structures in APS–substantia innominata (SI).

**Method:**

The presentation follows various steps: (1) location and boundaries of the zone and its neighboring areas; (2) schematic description of the region with simple outlines; (3) cursory revision of the SI and its three systems; (4) serial images of the dissections of the zone and its vessels, illustrated and completed when possible, by MRI images of a voluntary experimental subject (ES).

**Results:**

With this method, we could expose most of the structures of the region anatomically and imagenologically.

**Discussion:**

The zone can be approached for dissection with magnification and the habitual microsurgical instruments with satisfactory results. We think that fibers in this region should be followed by other anatomical methods in addition to tractography. The principal structures of ventral striopallidum and extended amygdala (EA) can be identified with the SPACE sequence. The amygdala and the basal ganglion of Meynert (BGM) are easily confused because of their similar signal. Anatomical clues can orient the clinician about the different clusters of the BGM in MRI.

**Conclusions:**

The dissection requires a previous knowledge of the zone and a good amount of patience. The APS is a little space where concentrate essential vessels for the telencephalon, “en passage” or perforating, and neural structures of relevant functional import. From anatomical and MRI points of view, both neural and vascular structures follow a harmonious and topographically describable plan. The SPACE MRI sequence has proved to be a useful tool for identifying different structures in this area as the striatopallidal and EA. Anatomical knowledge of the fibers helps in the search of clusters of the basal ganglion.

## Graphical abstract

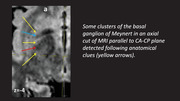

## 1 INTRODUCTION

In 2002, we presented an article on the anterior perforated substance (APS) to the Congress of the Asociación Argentina de Neurocirugía (Fontana et al., 2002). We described a series of microdissections of the zone and its vessels, compared them with images of the atlas of Foix and Nicolesco (1925) and De Armond et al. (1978) and made a cursory revision of the complex functional structure of this region. As expectable for practicing neurosurgeons, it was the result of a discontinuous but perseverant work, in a span of years at the Hospital Central de San Isidro. The article had a good reception in our community.

Since then, the knowledge of the region and its importance for the symptomatology of different neurological, neurosurgical, and psychiatric processes has grown enormously. Recently, we had the opportunity to access new anatomic material and also to discuss its imagenological anatomy in a facility offered by ARGUS Diagnóstico Medico San Isidro, in the valuable interaction with our colleagues at this Institution.

## 2 OBJECTIVE

Our objective is, to better describe the zone, combining our previous anatomical experience with the new one and, based on it, to analyze the anatomical potentialities of the MRI images obtained with the SPACE sequence. Our principal aims are, based on anatomical knowledge, MRI identification and topographical location of structures in the APS as widely as possible.

## 3 METHOD

We utilized part of the material prepared for our first article: 12 anatomical specimens, 10 of them exposing the vasculature of the region. In addition, in the other four hemispheres, images of the progressive dissection of the zone with the technique described by Klingler and Gloor (1960) for the preparation of fibers. Now we add two hemispheres to the series of dissections. One of them was already coronally cut 2 cm behind the level of the mammillary bodies, preserving in the anterior half, the whole region object of our study (it is our “Charcot's cut hemisphere”).

The dissection was done under a Newton Op wall microscope or sometimes, with 2.5× loupes and with the habitual microsurgical instruments. When the microscope was not used, the photo camera was a Sony DSC‐HX80 with lens and Zeiss Vario‐Sonnar T* 3.5‐64/4.1‐123 camera. We neither used an aspirator nor irrigat**i**on during the time of dissection, which in the last time, was always short. It lasted about one or one hour and a half each time. During the work, the tissue loses progressively its moisture and achieves a state that favors the fiber preparation. This ideal period lasts approximately half an hour in our experience or maybe a little more, depending on the ambient conditions. The pictures can be obtained both at the end of this period or at the beginning of the next session.

The approach of the dissections presented was, with few exceptions, preferentially from below and from the inside, which allows better appreciation of the different planes of the region, and their respective deepness. The anatomical findings were correlated with histological pieces of various atlases: Foix and Nicolesco (1925), De Armond et al. (1978), and Mai et al. (2016) and compared with classical and actual descriptions of the zone. We analyzed the MRI images of a 17‐year‐old normal female subject (experimental subject [ES]), with the SPACE sequence: turbo spin echo, single‐slab 3D T2 dark fluid, TR: 5000 ms, inversion time: 1800 ms, echo time: 387, echo train length: 246, slice thickness 1 mm in an MRI Skyra 3T, Siemens Medical Systems. This is an isotropic 3D sequence; therefore, the voxels generated by the 3D acquisition measure the same in each direction, allowing the images to be reformatted with equal resolution in any direction. Besides, it has very long echo train lengths, typically 100–250 echoes, ultrashort echo spacing, and nonselective refocusing pulses. The advantages of this technique over conventional two‐dimensional FLAIR have been demonstrated for several indications, including multiple sclerosis, brain tumors, and endolymphatic hydrops, mainly because it provides thinner slice thickness, lower partial‐volume effect, higher lesion contrast‐to‐noise ratio, and reduced flow artifacts. This sequence was selected because, in our opinion, it allows the better differentiation of tissues in the narrow space of the zone. The images were stripped of the cranium and analyzed in 2D and 3D, coronal, axial, and oblique plans, selected for the better visualization of the structures, with the BioImage Suite program (Papademetris et al., 2006). The parameters of contrast (minimal and maximal intensity) were set always at the same values. Stereotactic coordinates were calculated with the method of Mai et al. (2016). Measurements were done exceptionally with Radiant DICOM Viewer Medixant (64 bit).

Finally, we will revise succinctly the structure of the substantia innominata (SI) and the important progress that MRI has signified for its knowledge.

The order of presentation of the results will be as follows: (1) the APS location and limits; (2) the schematic description of the structures that will be exposed by dissection; (3) a brief revision of the three systems of the SI and their functions; (4) anatomical and MRI analysis of the SI in its fiber and cellular systems. Great part of the text of Section 4 is limited to a commentary of each image. We think that when the subject is complex, better than a description is an outline or a good image. The figures are thematically arranged.

## 4 RESULTS

### 4.1 Boundaries

**FIGURE 1:**
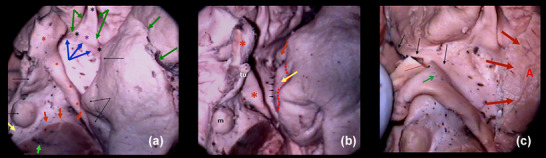
**Boundaries** (from our old series) (left hemisphere). **(a) lilac and black stars,** olfactory tract and internal and external olfactory striae; **red stars,** optic chiasma and tract; **black delicate arrows,** hippocampal uncus. The **green arrows** demonstrate the complexity of the rhinal fissure in the human; **blue arrows,** entorhinal fissure. Also indicated, tuber and mammillary body (**delicate arrows at the midline**), cerebral peduncle (**red arrows**), substantia nigra (**light green arrow**), and posterior perforated space (**yellow arrow**). **(b) Medial point of view**—**little fine arrows,** entorhinal fissure along the semilunar gyrus of uncus (**yellow arrow**); **red arrow,** falciform fold; **red dashed line,** semianular sulcus; **red asterisks,** chiasma and optic tract; **tu,** tuber; **m,** mammillary body. **(c)** Optic chiasma and tract reclined downward and inward to expose the medial portion of the anterior perforated substance (APS)—**thin arrows,** diagonal band of Broca (Broca, 1879) (**dbB**) in its horizontal and vertical limbs. The band does not present perforations in its course. Thus, many authors consider it constitutes the true posteromedial limit of the anterior perforated substance. The lamina terminalis is torn and not visible. Observe perforations over the union between chiasma and optic tract (OT), in the preoptic area (**green arrow**); **red arrows,** superior lip of the opening of the semianular sulcus during dissection. Once transposed into the cortex, the rest of the amygdala (**a**) can be exposed. The lateral portion of the APS is still hidden by the amygdala.

The shape of APS is roughly rhomboid, and its limits are as follows: forward the olfactory trigone and the external and internal olfactory striae; inward the optic tract and the lateral border of the optic chiasm; outward the anterior extreme of the hippocampal gyrus and its uncus (Figure 1a).

The APS seems to extend in anteroposterior sense, but its internal part is hidden by the chiasm and the external part by the hippocampal uncus. Thus, its transverse diameter results in being the longest. The zone is in humans and primates, clearly depressed with a deepness of about 5–10 mm with respect to the posterior orbital cortex. Contrarily, in other mammals, it appears as a salience more or less pronounced: *the olfactory tubercle*. From the inside, it can be seen that the entorhinal fissure when visible is the true limit of the APS, alongside the semilunar gyrus of the uncus (Figure 1b). The APS ends its lateral extension at the falciform fold. The uncal semianular sulcus delimits the semilunar gyrus (amygdala), from the rest of the uncal cortex. We entered this sulcus to separate the rest of the temporal lobe from our region, during dissection (Figure 1c).

### 4.2 Medial surface of the brain and septum

**FIGURE 2:**
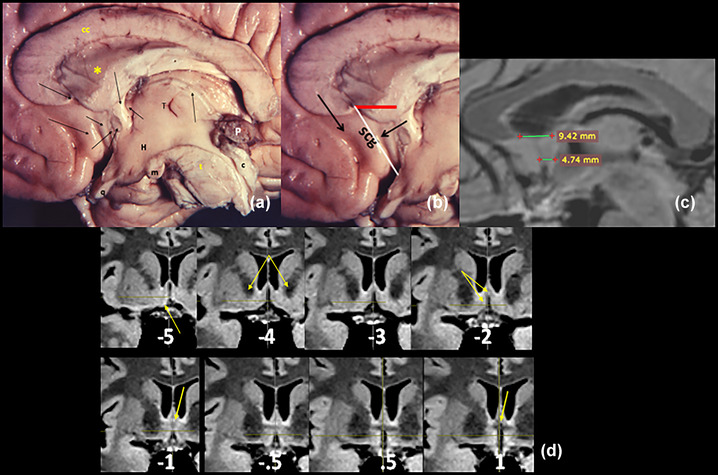
Medial structures intimately related to the anterior perforated substance (APS). **(a) cc,** corpus callosum; **star,** septum lucidum with anterior septal vein; **first horizontal row of black arrows,** left to right—beak of the corpus callosum, poscommissural fornix, foramen of Monro, stria medullaris; **second row**—subcallosal gyrus (**scg**); paraterminal gyrus or vertical limb of Broca's band (**vdbB**); lamina terminalis; anterior commissure (**ac**). **H,** hypothalamus; **q,** optic chiasm; **m,** mammillary body; **t,** mesencephalic tegmentum; **c,** collicular plate; **P,** pineal gland**. (b)** Anterior and posterior paraterminal sulci— **white line,** anterior limit of the septum. **(c)** MRI image from our model measured with Radiant DICOM Viewer. **(d)** Coronal cuts are better for analyzing the septum. In our model, a run of slices perpendicular to the CA–CP plane from 5 mm ahead of the ca, till 1 mm behind it. In the last slice (right below), the triangular recess of the III V (**yellow arrow**); in −0.5 and −1, crossing of the vdbB in front of the ca; in −5, beginning of the scg (**yellow arrow**). Ahead of there, the fissure becomes progressively closed by the thick scg. In −2 **yellow arrows,** possible Ch1 and Ch 2 nuclei. Ch 1 seems to prolong itself till −0.5. In all the slices in the inferior row, the presence of the hypothalamus‐preoptic region makes the septum to be only pre‐ and supracommissural.

With data from Andy and Stephan (1976), we can see in the **red bar**, the major A–P extension of the *septum verum* (the thickest portion of the septum, to distinguish it from the *septum pellucidum*, the thinnest one) in human. The anterior limit is a line that grossly follows the posterior paraterminal sulcus from the beak of the corpus callosum. The septum verum involves a minimal part of the posterior superior subcallosal gyrus (**scg**) and the whole paraterminal gyrus (Figure 2). Together with the commissures, this zone constitutes the *septo‐commissural area*.

For some authors, the appearance of the pallidum in a series of coronal cuts indicates the beginning of the septum (Butler et al., 2014). For us, the broad interhemispheric fissure between both vdbB indicates we are cutting in the septum till 5 mm ahead of the ca, which marks the beginning of the subcallosal gyrus, and the fissure progressively disappears. The triangular recess of the III ventricle indicates the posterior end of the septum (Butler et al., 2014) (Figure 2).

### 4.3 Outlines

**FIGURE 3:**
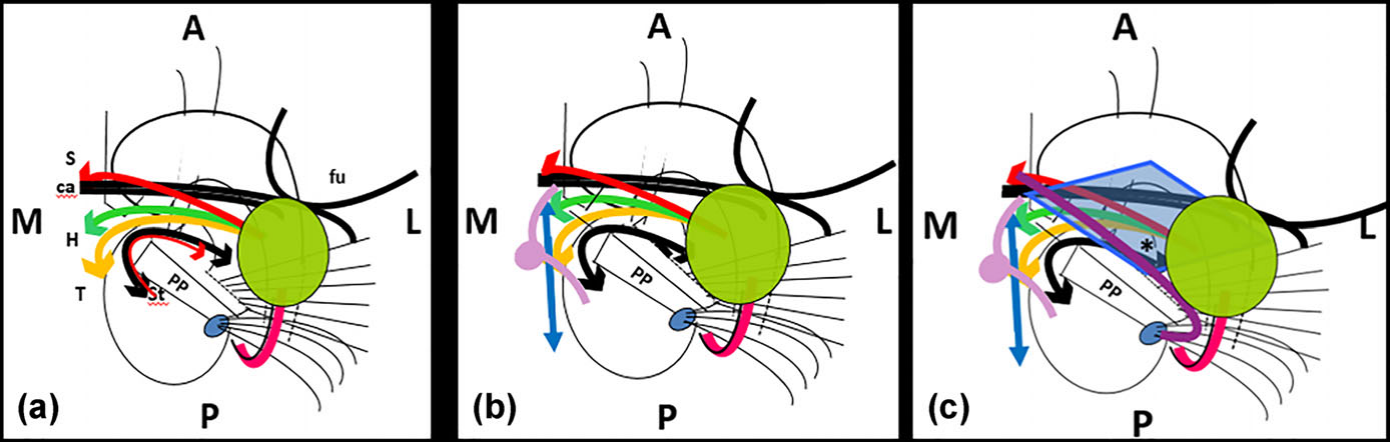
**Ansa peduncularis (Ap) and medial forebrain bundle (MFB)** (26 redesigned). Over an outline of the internal capsule and the basal ganglia viewed from below**, the anterior commissure** (ca) **and the unciform fascicle** (fu) are the deepest (highest with the brain in normal position) structures of the region**. (a) Red,** diagonal band of Broca, horizontal limb (hdbB)**; green,** amygdalo hypothalamic**; orange,** inferior thalamic peduncle**; black and red arrows,** ansa lenticularis. **(b) Blue bidirectional arrow,** the *medial forebrain bundle* **(MFB)** (**Nieuwenhuys et al.,** 1982), passes through the lateral hypothalamus, between the ansa and the fornico‐mamilo‐thalamic plane **(lilac)**, in an approximately horizontal track. **S,** septum; **H,** hypothalamus; **T,** thalamus; **Sth,** subthalamic nucleus. **(c) Approximate limits of the anterior perforated substance (APS)**. The amygdala **(green**) hides the lateral portion of the APS **(light blue)**. The optic tract and posterior part of the optic chiasm (**purple**) separate the region from midbrain and tuber. Note the relation among **optic tract, cerebral peduncle, and amygdala. Asterisk,** triangular zone under the pallidum to be discussed in the section of the basal nucleus of Meynert.

Jhonston (1923) described for the first time a system of fibers that traverse the zone and denominated them *the X component*. Later, it was named ***ansa peduncularis*** (**Ap**) in association with ***ansa lenticularis*** (**Al**). The Ap is formed from depth to surface and posterior to anterior by Klingler and Gloor (1960) (**see** Figure 3): (1) *the inferior thalamic peduncle* (**IthP**), which ends in the midline and MD thalamic nuclei. It carries fibers from amygdala, temporal and insular cortices, tuberculum olfactorium, and ventral pallidum (VP). (2) *The amygdalo hypothalamic fibers (Ah*) end in nuclei of the medial and lateral hypothalamus. (3) The *amygdalo septal* fibers collect in but are not the unique component of the diagonal band of Broca (Sakamoto et al., 1999) *(dbB)*. All these fibers are also named *the ventral amygdalofugal way (* ***VafW***). (4) **The (Al)** coming from the pallidum toward Forel's field encircles the anterior border of the internal capsule just before it becomes the cerebral peduncle (**Figures** 8, 11, 12, 13 **e,f**). There are bidirectional connections between nucleus subthalamicus (Sth)‐–xternal pallidum (**Pe**) (**red** in Figure 3 left) but only **afferent** for the Pi. This component of the tract is named ansa subthalamica (Lopes‐Alho et al., 2019). It is replaced in the rest of the series for a short black line. The AL is adherent to the internal capsule. Near the midline, all these fibers change direction to be parallel to the sagittal plane aiming to their respective end areas. Thus, they received the name of “ansa” (Figure 3). The vertical limb of Broca's band constitutes what the classic anatomists named columns *or pillars of the septum* (Edinger, 1904). Its fibers confound with the *precommissural fornix* (**pcf**).

### 4.4 The anterior limb of the internal capsule (ALIC)

**FIGURE 4:**
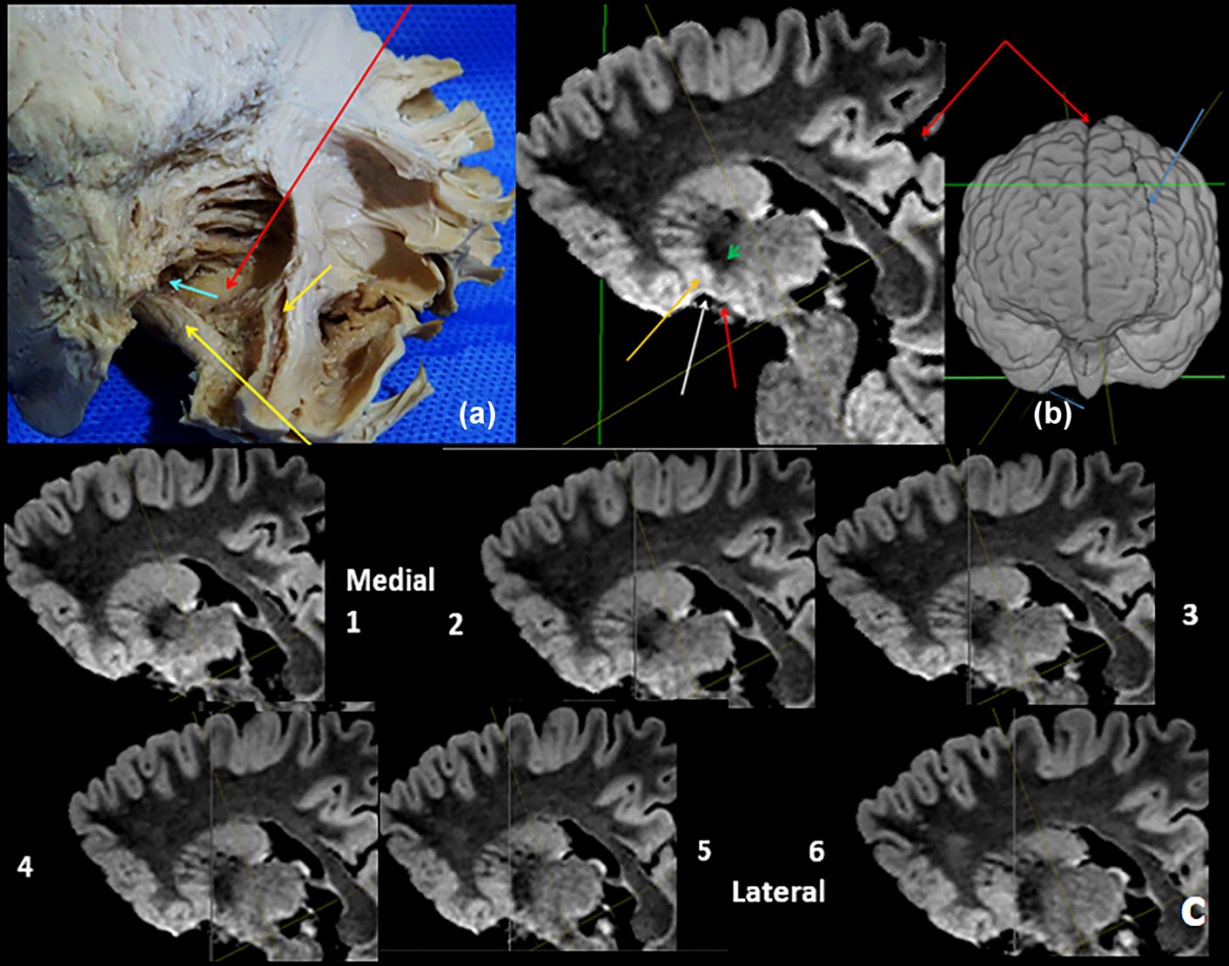
**(a)** Right hemisphere of Charcot's cut. External capsule and lenticular nucleus resected—**light blue arrow,** portal for the bridge accumbens putamen; **orange arrow,** furrow for the claustrum; **yellow arrow,** anterior commissure; **red arrow,** floor of the capsule. **(b)** Oblique MRI cut through the anterior limb in our model with an inclination of 35° in the coronal, and of 25° in the axial sense. The anterior limb is well shown—**white arrow,** medial anterior perforated substance (APS); **orange arrow,** accumbens; **red arrow,** optic tract; **short green arrow,** anterior commissure; **at the side,** oblique plane seen upon a 3D reconstruction of the brain of our model (**light blue arrows**); **red arrows,** sagittal fissure. See the difference of signal of the frontal and temporal poles with this sequence. **(c)** MRI in our model. A run of 0.5 mm oblique cuts of the anterior limb of the internal capsule; its breadth imports approximately 3.5 mm. *Note*: The greater intensity of signal of the accumbens.

The two portions of the internal capsule that form part of the region as its superior boundary are the anterior limb (ALIC) and its genu. Resecting the LN, we found the internal wall of its capsule, constituted by the ALIC, with its radiated fascicles and the spaces between them, through which pass the gray substance bridges that unite the head of the caudate nucleus and putamen; its inferior‐most fascicle contains fibers from the ventral anterior cingulate cortex and from the orbitofrontal cortex (Haber & Knutson, 2010; Haber et al., 2020) (but see [Hurwitz et al., 2006]). The lowest bridge of gray substance corresponds to the accumbens nucleus–lower putamen (fundus putamen [Mai et al., 2016]) (Figure 4 **above left**). We should be able to see the radiated arrangement of the ALIC in the tractographic reconstructions of its structure. The latero‐inferior wall is constituted of the fibers of the external capsule and the furrow in the unciform fascicle–IFOF–frontotemporal fascicle of the extreme capsule where the inferior border of the lateral claustrum fits. The anterior commissure passes below the extracted putamen. The floor of the capsule is formed by the external capsule at this level. Medially, the fibers of the beak of the corpus callosum also take part. The fibers of the ALIC concentrate just over the commissure. Thereafter they open up forward fanlike as radii (Figure 4b). The inferior border of ALIC determines the superior limit of the nucleus accumbens for many authors (Mavridis & Boviatsis, 2011; Neto et al., 2008; Salgado & Kaplitt, 2015). We think that in 1 and 2, of Figure 4c, the anterior peduncle of the thalamus (anterior thalamic radiation) is visible entering the ALIC *medially* to the rest of its fibers.

### 4.5 The substantia innominata (SI)

Name selected by Reil (Heimer & van Hoesen, 2006) at a time in which its composition and functions were unknown and was so maintained till the last four to five decades in which they were progressively discovered. As in every time, it only means the name of *a region* of the brain. The APS is the surface of a plate of gray substance of around 1 cm of thickness, traversed by multiple fascicles, extended under the nucleus lenticularis (Crosby et al., 1962; Foix & Nicolesco, 1925) and the ALIC: *the SI*.

**FIGURE 5:**
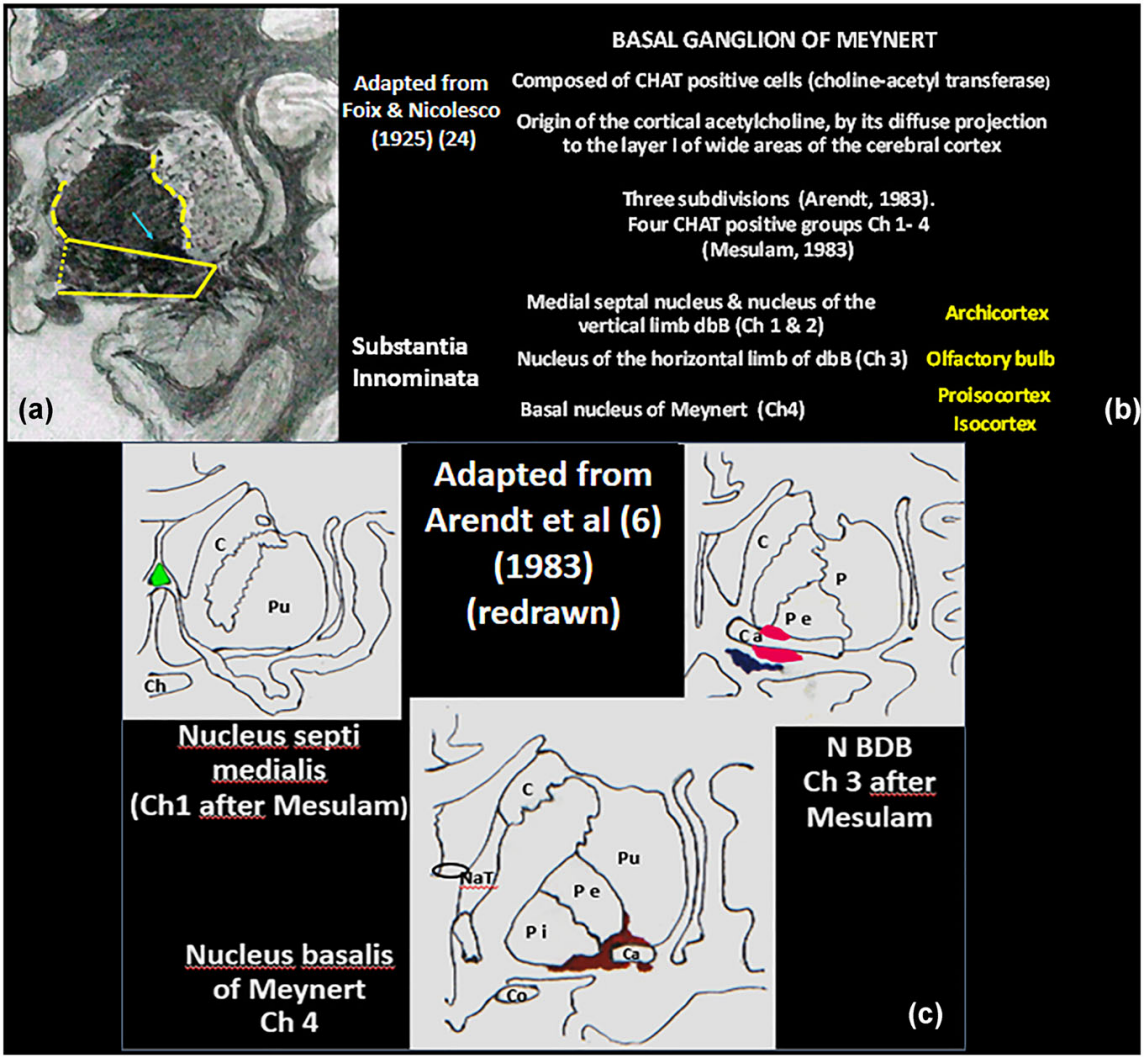
**(a) The *region* of the substantia innominata (SI)** demarcated in a coronal cut adapted from Foix and Nicolesco (1925) at level of the foramen of Monro and genu of the internal capsule—**fine dashed line,** artificial separation from hypothalamus; **gross dashed lines,** extensions of the basal ganglion to the external medullary lamina of the lenticular nucleus and stria terminalis that can reach the internal capsule; **light blue arrow,** anterior commissure. **(b)** Abstract of the **basal ganglion. (c) Location of some cholinergic nuclei of the SI**—Mesulam's group Ch1 (medial septal: **green**) and nucleus of the vertical diagonal band (Ch2) are septal; Ch3 or nucleus of the horizontal band (**blue**) is next to the principal portion of ventral pallidum painted in **red**; anterior Ch4 extends to the external medullary lamina of the lenticular nucleus. **C,** caudate; **Pu,** putamen; **Ch,** chiasma; **Pe,** external pallidum; **Pi,** internal pallidum; **Co,** optic tract; **NaT,** anterior nucleus of the thalamus; **oval,** foramen of Monro; **Ca,** anterior commissure. *Source*: (c) Adapted from Arendt et al. (1983).

Three intertwined systems make up the SI: *the basal ganglion of Meynert (* ***BGM***), *the extended amygdala (* ***EA***), and *the ventral striatum (* ***VS*** *) and* ***VP***. The region extends between a plane passing through the anterior commissure/inferior border of the ALIC and the surface of the APS (Liu et al., 2015). In a volume of tissue of approximately 1 cm high are arranged the system of fibers described above plus the three mentioned systems of grey substance. These cell systems are, till now, only identified cytochemically and by their different connections but are intricately mixed spatially (Foix & Nicolesco, 1925; Haber et al., 1993; Heimer & van Hoesen, 2006; Heimer et al., 2008; Liu et al., 2015) (Figure 5).

Moreover, they exceed the area of APS on all of its sides, though it could be considered to be at its center. These features dismiss any macroanatomical essay to investigate the grey substance of SI but would allow some possibility to the images.

The **VS** is constituted by a nucleus approximately limited: the nucleus accumbens, below the head of the caudate and the anterior inferior putamen, both below the level of the inferior border of ALIC/anterior commissure. The accumbens is medial to a vertico‐sagittal plane from the ALIC, the ventral putamen lateral to it; the VS is also constituted of groups of cells that extend irregularly backward “as fingers” intermingled with the VP and EA (Haber et al., 1993; Heimer & van Hoesen, 2006; Heimer et al., 2008). Although conventionally accepted as the superior limit of the VS, “it is not possible to precisely identify boundaries separating any of these structures from each other” (Heimer & van Hoesen, 2006).

The **VP** has a typical place below the anterior commissure when it passes through the external pallidum and also in a little extension, over it, in the inferior‐most portion of the external pallidum (Figure 5c). Posterior, there are little zones under the pallidum that also belong to the VP (Haber et al., 1993).

The **BGM** was best described by Mesulam et al. (1983) (Arendt et al., 1983; Ayala, 1915; Brockhaus, 1942; Engelhardt, 2013; Haber et al., 1993; Hedreen et al., 1984; Jones et al., 1976; Liu et al., 2015; Mesulam & Ch, 1988; Mesulam et al., 1983, 1984, 1986; Page & Sofroniew, 1996; Sakamoto et al., 1999; Simic et al., 1999; Tenenholz‐Greenberg & Heinsen, 2007). Two portions (Ch1 and Ch2) are septal, Ch1 at the medial septal nucleus, and Ch2 in the vertical limb of the diagonal band of Broca. Both nuclei connect with the hippocampus (archicortex). Ch3 is in the horizontal limb of the dbB and connects with the olfactory bulb. Ch4 is the true BGM. It projects onto the rest of the cerebral cortex. The septal Ch1 and 2 nuclei are the origin of the whole acetylcholine content of the hippocampus and through this mechanism have an important role in memory (Alonso et al., 1996; Mesulam & Ch, 1988; Mesulam et al., 1984; Teles‐Grilo Ruivo & Mellor, 2013). The cholinergic projection to the hippocampus is topographically organized (Teles‐Grilo Ruivo & Mellor, 2013).

The septal nuclei in general have a theta electrographic rhythm self‐paced or maybe dependent on the reticular formation of the brain stem (O'Keefe & Nadel, 1978). This rhythm is transmitted by the precommissural fornix (**pcf**) to the hippocampus and through it, to the temporal lobe and may be, to other areas of the cortex.

On the other way, the Ch4 nucleus provides acetylcholine to the rest of the cortex through broad topographical connections (Mesulam & Ch, 1988; Mesulam et al., 1984). These connections are reciprocal only with limbic and para‐limbic areas (Mesulam et al., 1983) and are also predominant in them, with respect to the neocortex.

We think the outlines displayed in Figure 5 are useful anatomical clues to orient the image analysis.

#### 4.5.1 Extended amygdala: the field of the bed nucleus of the stria terminalis (BNST)

**FIGURE 6:**
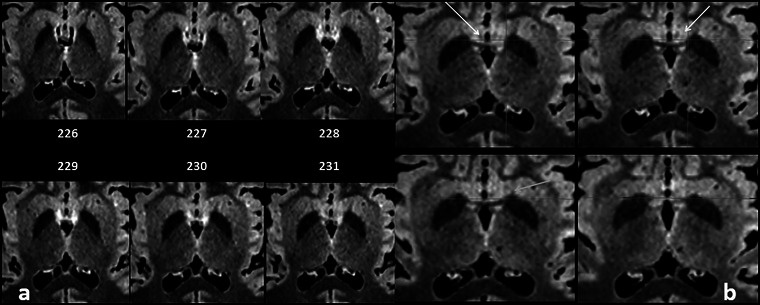
(a) A run of 0.5 mm slices from the deepest portion of the frontal horn down to the anterior commissure—Slice 226, foramen of Monro; Slices 229 and 230, bed nucleus of the stria terminalis (BNST) traverses the midline below the ac with some fibers of the St. (b) Another four downward slices around the anterior commissure; below it, the BNST diminishes as the post‐commissural fornix penetrates in the hypothalamus—**white arrow,** precommissural fornix and septum; **yellow arrow,** probably the anterior limb of the anterior commissure; **orange arrow,** inferior border of anterior limb of internal capsule.

**FIGURE 7:**
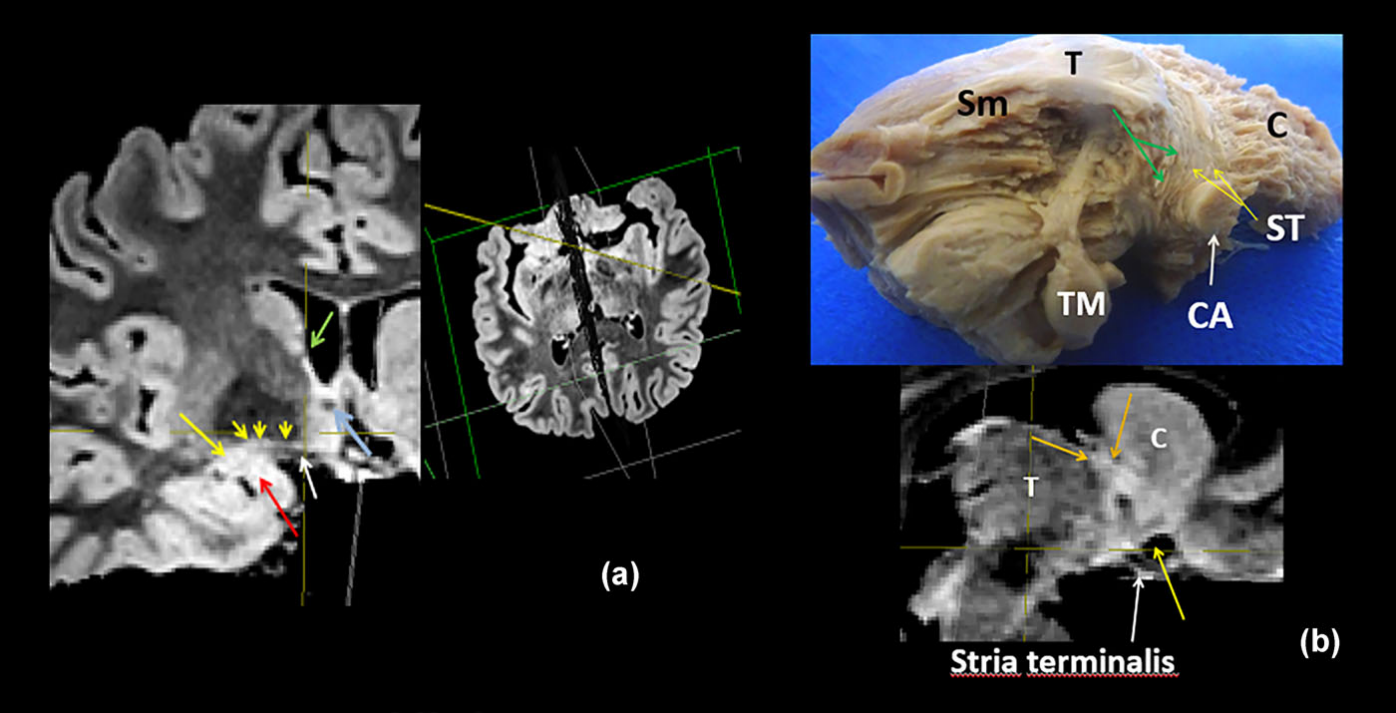
**(a)** MRI oblique plane of cut in our model to expose the continuity of **the sublenticular extended amygdala** (**SLEA**), from the centro‐medial amygdala to the bed nucleus of the stria terminalis (BNST). The obliquity of the cut is shown at the side—**bigger yellow arrow,** central nucleus of the amygdala; **red arrow,** medial nucleus of the amygdala; **little yellow arrows,** sublenticular extended amygdala with characteristic irregularity/discontinuity in human (Heimer et al., 2008); **white arrow,** optic chiasm and optic tract. The cut is almost parallel to them, but tilted posteriorly; **light blue arrow,** anterior commissure encircled by the BNST; **green arrow,** thalamostriate vein; extending downward from it, the dorsal bed nucleus (BNSTd). **(b) Above**—Fibers of the stria terminalis (ST) arrive at the region of the bed nucleus following the thalamo‐striate sulcus and divide in its pre‐ and postcommisural components (**yellow arrows)**. They are accompanied by the supracapsular (dorsal) portion of the bed nucleus (BNSTd). **T,** thalamus; **Sm,** stria medullaris; **TM,** mammillary body; **C,** caudate nucleus; **CA,** anterior commissure; **ST,** stria terminalis. The stria terminalis and stria medullaris interlace their fibers around the commissure (**green arrows**) (Crosby et al., 1962; Jhonston, 1913). **Below**—MRI sagittal cut. The bed nucleus distinguishes for its high signal and seems accompanied by fibers of the pre‐ and post‐commissural components of the stria terminalis. **white arrow,** optic chiasm; **yellow arrow,** medial anterior perforated substance (APS); **Orange arrows,** stria terminalis pars dorsalis.

The bed nucleus of the stria terminalis (**BNST**) forms the part of the **EA** and is located around the anterior commissure, medial to the genu of the internal capsule (Alheid, 2003; Avery et al., 2016; Haber et al., 1993; Heimer et al., 2008; Mai et al., 2016); it reaches the lateral face of the anterior pillar of the fornix medially, the anterior nucleus of the thalamus posteriorly, and extends forwards to the septum lying lateral and ventral to the lateral septal area. (Figure 6a,b). **BNST** has as characteristic the highest intensity signal, more than amygdala, and other limbic structures in this sequence inclusive more than in other FLAIR sequences (Schneider & Vergesslich, 2007; Theiss et al., 2017). The BNST is an extensive formation that encircles the whole internal capsule alongside and medial to the caudate. From the dorsal (supracapsular) BNST (BNSTd) descends over the anterior pole of the thalamus to the BNST, closing the circle, from the centro‐medial amygdala nuclei backwards, till the BNST and from there, through SLEA, again to the centro‐median nuclei. (Figure 7a,b).

The concept of the EA was found by an Argentine researcher: José Santiago de Olmos (Heimer et al., 2008), who created a cupric‐silver histochemical method that makes visible its structures.

### 4.6 Dissections

#### 4.6.1 Ansa peduncularis (see also Figure 3)

**FIGURE 8:**
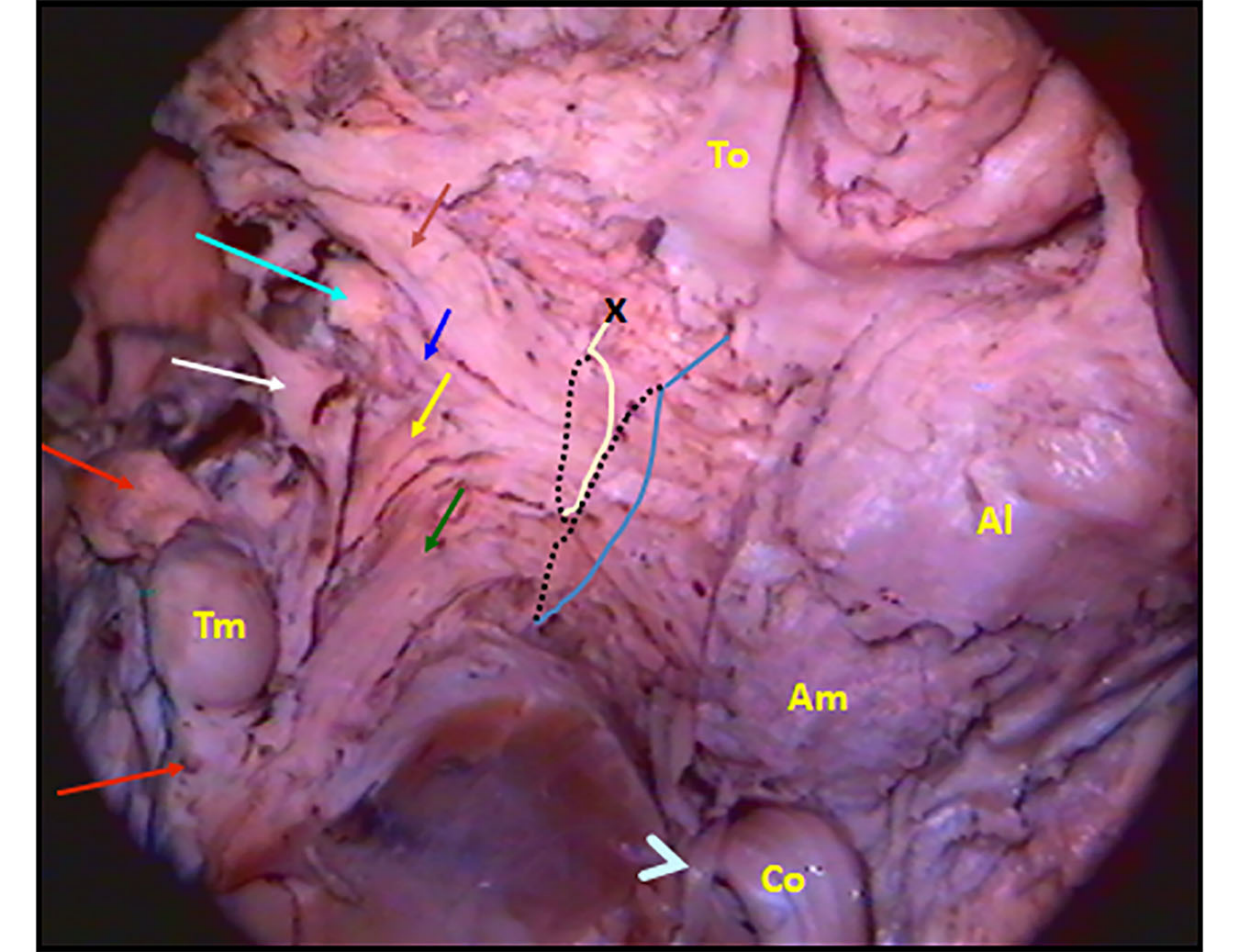
**Ansa peduncularis (see also** Figure 3) (left hemisphere). From front to back—the diagonal band (**fuchsia little arrow**), the amygdalo hypothalamic fibers **(blue**), the inferior thalamic peduncle (IthP) (**yellow**), and the ansa lenticularis (**green**) (Klingler & Gloor, 1960). **Al,** basolateral amygdala, with intraventricular brilliant part; **Am,** posteromedial part higher positioned. **Co,** optic tract reclined with a sulcus in its exposed face, where Meynert's commissure fits **(light blue arrowhead**) (Foix & Nicolesco, 1925). **Commissura anterior** (**light blue arrow**); **white arrow,** medial forebrain bundle MFB free in its anterior end, passing between the fornico‐mamillo‐thalamic plane (**Tm** and the **two red arrows**: the anterior signals the fornix, the posterior one, the mammillothalamic tract). **To,** arrival of the olfactory tract at the zone. **X oval** embraces the ventral amygdalofugal pathway. The bigger one embraces the whole ansa peduncularis. *Source*: From our old series.

In Figure 8, a delicate peeling of the surface of the APS exposes the fibers of the whole ansa peduncularis as described in Figure 3b,c, especially in this specimen, because the region has a good A–P extension and this allows an adequate display of them. To preserve the amygdala in place, the approach was made by opening the semianular sulcus (Duvernoy, 1998).

#### 4.6.2 The diagonal band of Broca (1879) and the precommissural fornix

##### 4.6.2.1 Diagonal band of Broca (1879) (dbB)

**FIGURE 9:**
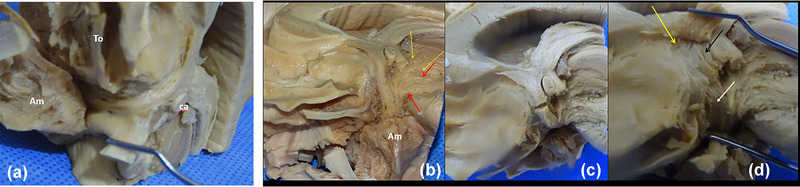
**(a)** Right hemisphere. View from below and medial. Optic nerve and chiasm reclined. **Am,** amygdala. **To,** olfactory tract; **ca,** anterior commissure. Both limbs of the diagonal band are visible. **(b)** Optic way and fornico‐mammillothalamic plane resected. The diagonal band is exposed in its two limbs. Exposed but not individualized, the inferior thalamic peduncle (IthP) (**yellow arrows**) and the ansa lenticularis (Al) (**red arrows**). Next to the amygdala (**Am**), the fibers form only one fascicle; medially they separate into three branches. **(c)** The vertical limb of diagonal band is cut. **(d)** The two limbs are reclined. The inferior limb is a compact fascicle that largely seems to come from the amygdala. The continuity of the two segments does not seem secured because the superior part has lost a bit of compactness (*precommissural fornix*: **pcf**). The gray substance above the inferior limb: Ch3 group of BGM (angular zone of the dbB [Brockhaus, 1942]) (**white arrow**); **yellow arrow,** precommissural fornix fibers that aim to the septum verum; **black arrow,** fibers aim to the hypothalamus.

The thickness of the distinct structures of the zone differs in individuals and structures. In this case, the diagonal band (Figure 9a) is much more salient than in Figure 1c. Observe that perforations are in front or behind the dbB, but not *in* it.

##### 4.6.2.2 The precommissural fornix

**FIGURE 10:**
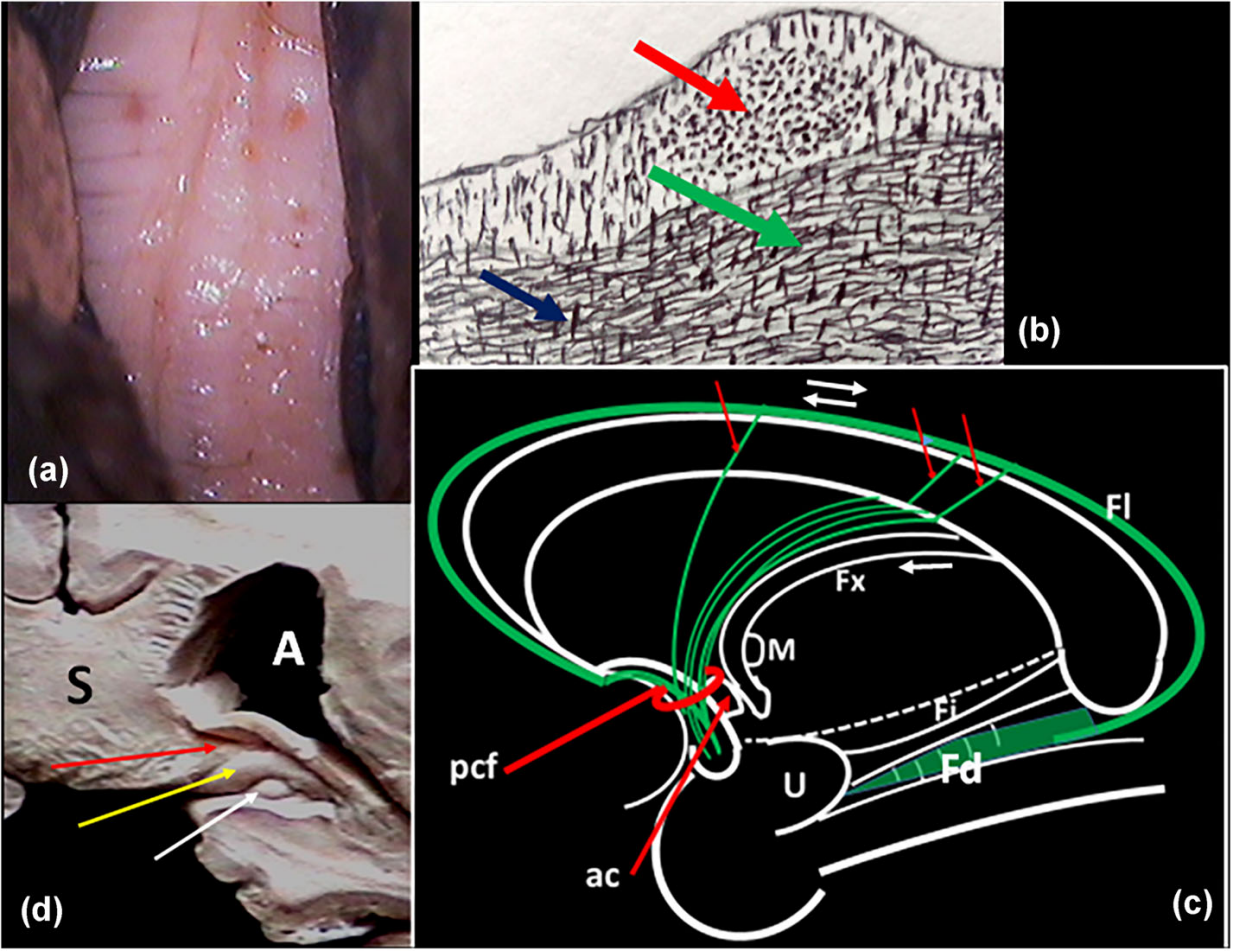
**(a)** The medial striae of Lancisi with an undulated course, a normal presentation (Pavlovic et al., 2009). **(b)** Midline coronal cut of part of the thickness of the human corpus callosum—**red arrow,** stria of Lancisi, the fibers of which travel perpendicular to the cut. There is an enormous group of vertical fibers that perforate the corpus callosum obliquely (see text). **(c)** Outline inspired in a figure of Jhonston (1913), representing a sagittal cut of the brain of a rat. Ours is supposed on man—**red arrows,** three perforator fibers that incorporate to the septum pellucidum, from the fornix longus; they (**green**) accumulate in the septum lucidum immediately dorsal to the fornix. **pcf,** precommissural fornix; **ac,** anterior commissure; **M,** foramen of Monro; **Fi,** fimbria; **Fl,** fornix longus; **U,** uncus; **dashed line,** cut of the upper brain stem to expose the medial temporal region; **Fd,** fascia dentate; **Fi,** fimbria. **(d)** Coronal cut at the level of the atrium—**red arrow,** fibers of the splenium (**S**), separate the fimbria from the fasciola cinerea (**yellow**); **white arrow,** Andreas Retzius’ gyri. *Source*: (b) Adapted from Kölliker (see text).

The striae of Lancisi are the continuation of a part of the hippocampal fimbria over the corpus callosum accompanied by a layer of atrophied cortex possibly from the fasciola cinerea (indusium griseum). They continue forward with the paraterminal gyrus in the septum. This system of the *fornix longus* was described by Von Kölliker (1894). The fibers of the striae perforate obliquely forward and downward the corpus callosum and unite below it, in the lamina of the septum pellucidum of the same side, arriving this way to the precommissural fornix. Some of them do not perforate and reach the pcf around the knee of cc. Kölliker thought those fibers would come from the cingulum (Ungerstedt, 1971) (green and blue arrows in part [B] of Figure 10). The design in Figure 10b is from him (1896), and we have redesigned it from a reproduction in G Mingazzini's “Der Balken” (1925) (Mingazzini, 1922). Jhonston (1913) had a similar concept (Figure 10 **c)**. Currently, this description seems forgotten or discarded and not replaced (Pavlovic et al., 2009). Recently, Cho et al. (2013) believed to have discovered “a new septal tract.” But they do not mention the interpretation here exposed. The fibers of the pcf diverge in two different directions (Figure 9d): *to the septum* (**yellow arrow**) and *to the hypothalamus* (**black arrow**). Eventually, some fibers follow the inferior limb to the APS *(olfactory tubercle)*. Other authors sustain that pcf is a branch of the anterior pillar of the fornix (Duvernoy, 1998). Possibly, the three described mechanisms are in play.

#### 4.6.3 Inferior thalamic peduncle (IthP) and ansa lenticularis

**FIGURE 11:**
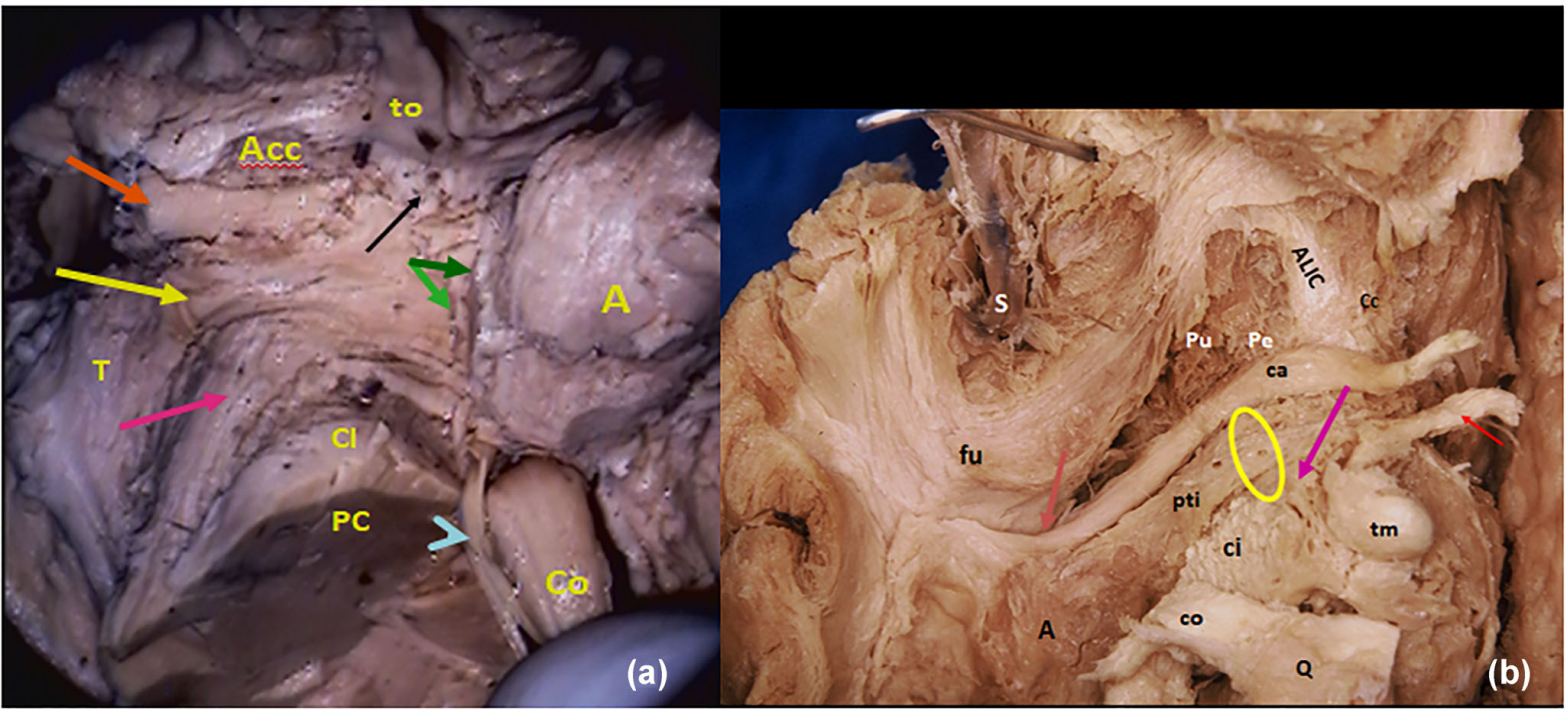
**(a)** (From our old series) **Progression of dissection of the left anterior perforated substance**. Diagonal band and amygdalo hypothalamic projection were resected. **to,** olfactory tract; **A,** amygdala; **Co,** optic tract reclined; **Arrowhead,** Meynert's commissure; **CI,** internal capsule; **PC,** cerebral peduncle; **T,** thalamus. The following planes can be discriminated from the surface—(1) anterior perforated substance (APS) surface with perforations (**black arrow**); (2) accumbens nucleus (**Acc**); (3) amygdalo septal and amygdalo hypothalamic ways resected (**green arrows**); (4) inferior thalamic peduncle (IthP) (**yellow arrow**); (5) ansa lenticularis (**red arrow**); (6) anterior commissure (**orange arrow**). The IthP overlaps in part with the ansa lenticularis, in its horizontal track. **(b)** (From our old series) Right cerebral hemisphere. **S,** sylvian fissure; silvian artery reclined; **ALIC,** inferior border of anterior limb of the internal capsule, after resection of the ventral strio‐pallidum; **Pu,** putamen; **Pe,** external pallidum; **Cc,** head of caudate nucleus; **fu,** unciform fascicle, **ca,** anterior commissure exposed from the midline; **pink arrow,** characteristic curling of the anterior commissure; behind and slightly below, but in contact with the commissure, travels the inferior thalamic peduncle (**pti**) that comes from the amygdala (**a**), covering in part the ansa lenticularis (**red arrow**). Observe the little holes of retro‐commissural arterial perforators. These holes can grow as Virchow–Robin spaces; **ci,** internal capsule; **tm,** mammillary body with anterior column of the fornix arriving (**short red arrow**); **co,** optic tract; **Q,** optic chiasm retracted.

Behind the olfactory trigon in primates, there can be a little elevation representing the tuberculum olfactorium (Sakamoto et al., 1999) of macrosmatics (Figure 11a)). In this specimen, the diagonal band was a very shallow strip (Figure 1c). It can be bigger, as in Figure 9a. Contrarily, the ansa peduncularis has a massive size. The IthP follows an almost right line till its change of direction, where it has a typical fanlike expansion that immediately opens before ending in the thalamus. The ca passes below and in contact with the ALIC, grubbing a canal at the inferior surface of the external pallidum, passing below the putamen, and emerging below and behind the unciform fascicle, at the temporal stem where it ramifies to the neocortex of the anterior temporal lobe. Their fibers show a characteristic curling “as a rope” (Klingler & Gloor, 1960) in this part of its course (Figure 11b).

**FIGURE 12:**
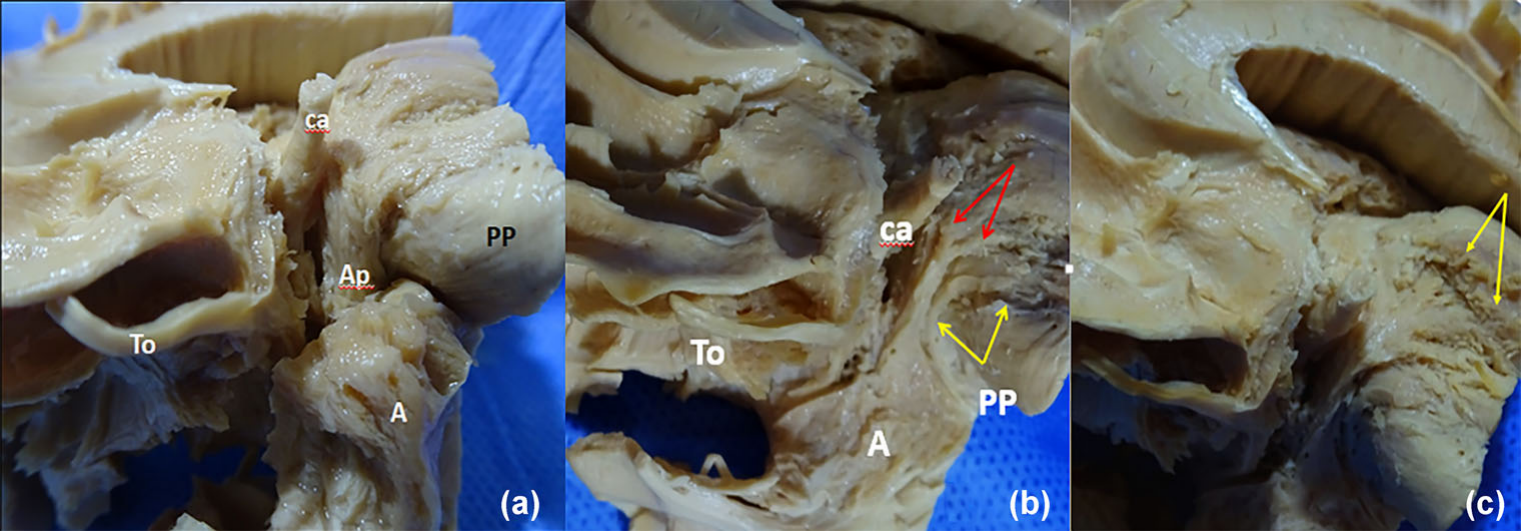
Progressive dissection of the inferior thalamic peduncle (IthP). (**a**) **ca,** anterior commissure with inferior thalamic peduncle and ansa lenticularis. Both structures carry most of the fibers of the ansa peduncularis (**Ap**). **To,** olfactory tract; **a,** amygdala; **ca,** anterior commissure; **PP,** cerebral peduncle. **(b)** The inferior thalamic peduncle (**red arrows**) where it opens in a fan on the medial and mediodorsal thalamus (**red arrows**). The ansa lenticularis encircles the cerebral peduncle (**yellow arrows**) with a strong bent. **(c)** The entrance of the inferior thalamic peduncle in the mediodorsal nucleus. **Yellow arrows** show more superficial points of ending, probably in midline nuclei.

Figure 12c shows the entrance of the IthP to the MD nucleus is 5–6 mm deep from the medial surface of the thalamus. Two cut lines of fibers penetrate in more superficial portions of the thalamus, probably in part in midline nuclei paraventricular (**Pv**) and paratenial (**Pt**) (Mai & Forutan, 2012). All these fibers come together in the IthP. Observe the gracility of structures with respect to the previous specimen.

**FIGURE 13:**
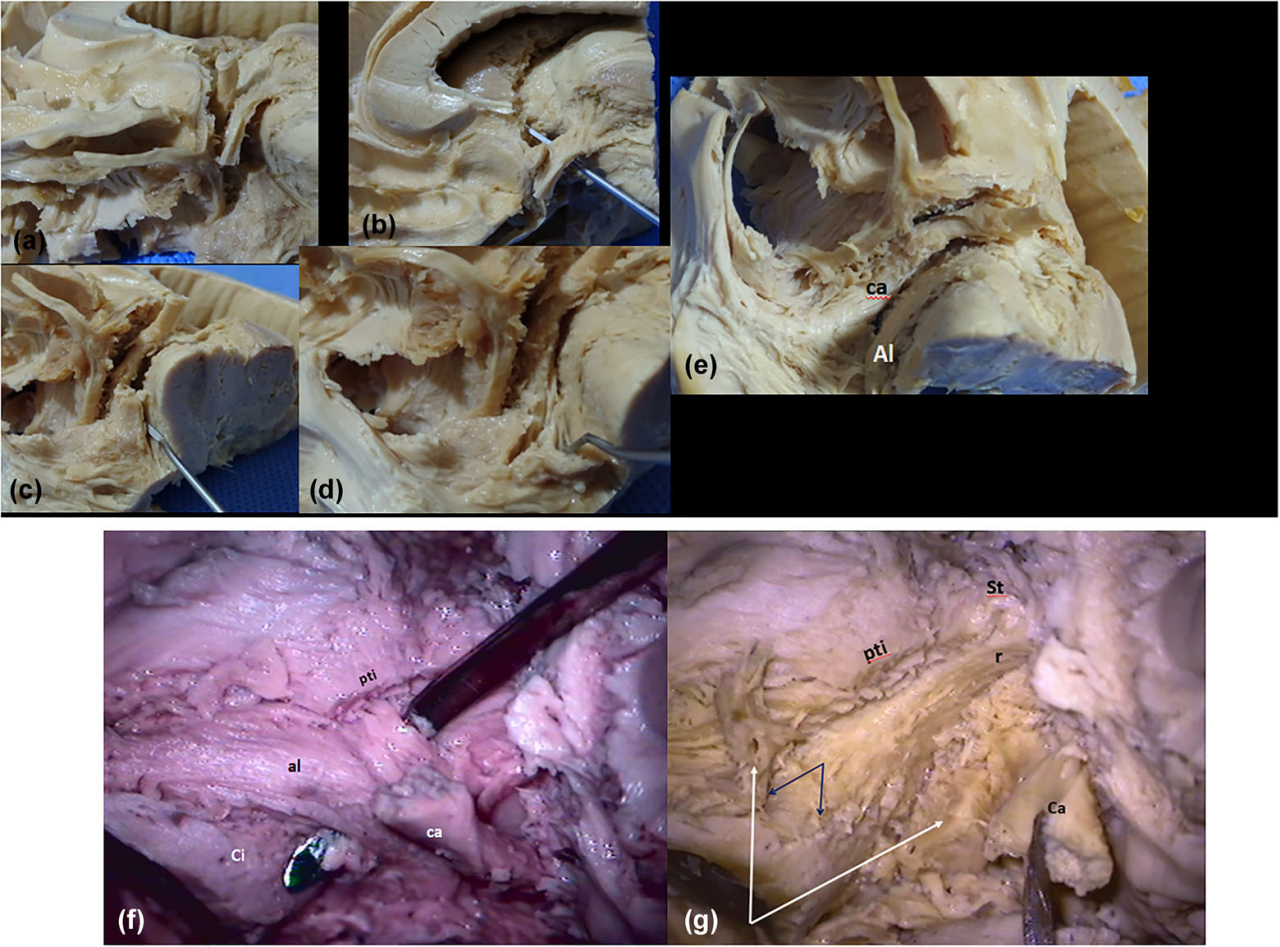
**Steps in the resection of the extracapsular thalamic peduncle (IthP): (a)** sectioning the inferior thalamic peduncle; **(b)** elevating and resecting its distal part; **(c)** dissecting the proximal part with amygdala; **(d)** reclining the amygdala, various fascicles of the anterior commissure can be seen ending in it**. (e)** After resection of the inferior thalamic peduncle, the ansa lenticularis is fully exposed (Al). **(f and g) Resection of the ansa lenticularis: (f)** The dissector passes between the ansa lenticularis (**al**) and the internal capsule **(Ci)**. Probably traverses the anterior pole of the subthalamic nucleus that is lowest at the level of the ansa. **pti,** rest of the resected inferior thalamic peduncle; **ca,** anterior commissure. **(g)** Lenticular ansa resected (**white arrows**) to see the internal capsule. **St,** end of the stria terminalis at the level of the genu of the internal capsule (**r**). Its pre‐ and post‐commissural sectors are insinuated; **Ca,** anterior commissure reclined at the beginning of Gratiolet's canal; **r,** genu of the internal capsule. **Blue arrows,** probable part of the location of the subthalamic nucleus, which we were unable to preserve. Observe at least two planes of fibers in the cut inferior thalamic peduncle. *Source*: From our old series.

The progressive resection of the ansa peduncularis as in Figure 13 allows a better comprehension of its relations with the internal capsule, just below the genu and posterior ALIC.

### 4.7 MRI of the ansa peduncularis

**FIGURE 14:**
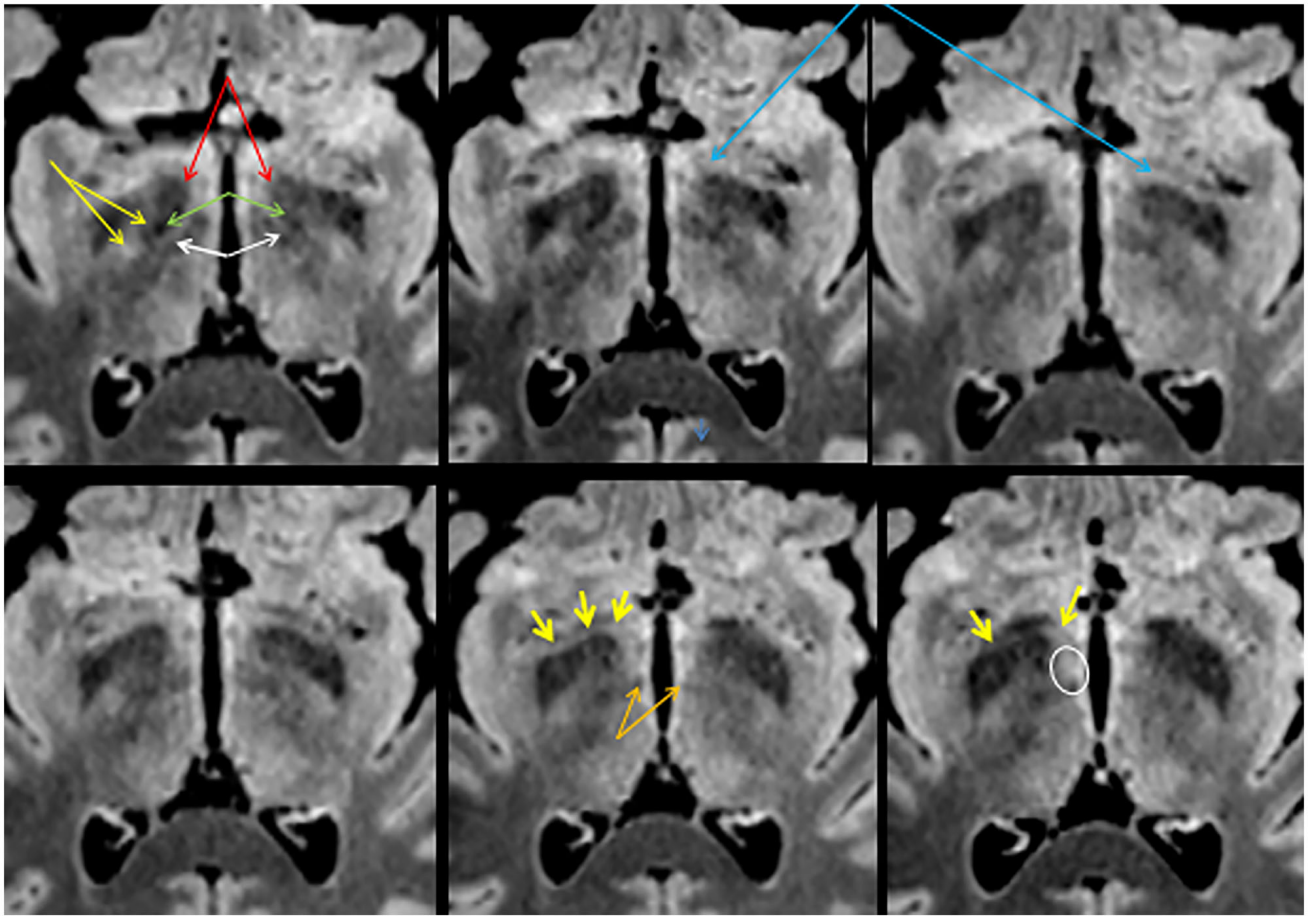
Axial upward series of slices 0.5 mm apart from 1 mm over the optic tract. Head in normal position—**yellow arrows,** internal capsule traversed by fibers of the lenticular fascicle; **red arrows,** ansa lenticularis; **green arrows,** lenticular fascicle; **white arrows,** thalamic fascicle. Between both is the zona incerta (Zi); **light blue arrows,** inferior thalamic peduncle; **orange arrows,** mammillothalamic tracts. (Compare with Figure 12c.)

The difference in signal intensity between the al and the IthP in this sequence (Figure 14) should be due to a different myelination of their fibers and maybe also, for the presence of a major cellular component around/between the fibers of the IthP (Schneider & Vergesslich, 2007). A thalamic lesion at the entrance of the IthP (**little yellow arrows** in the next to last figure of the inferior row of Figure 14) that also affects the mammillothalamic tract can produce severe memory troubles (Graff‐Radford et al., 1990) (**white circle** in the last figure). The arrangement of the structures is better visible on the right.

*Apparently*, in Figure 14, the path of IthP reaches the zone of the lateral hypothalamus and, from there, *seems* to continue in zona incerta in the first three slices. *Actually*, it ascends obliquely backward approximately from the very level of the heads of the red arrows in the first slice (Figure 12c).

#### 4.7.1 The medial forebrain bundle

The medial forebrain bundle (MFB) is a group of ascending and descending fibers that unite the septal region with the mesencephalic tegmentum with multiple stations on its path through the lateral hypothalamus. Like a sagittal wall, it separates the fibers of the ansa peduncularis that are changing their direction from horizontal to sagittal, on the one hand, and the fornico‐mammillothalamic plane on the other (Figure 15). These fibers take part in our zone of interest only in the sense that they constitute its deep medial border. We could find this structure in two of our dissections, and in both, it was impossible for us to find its origin, putatively in the septum (Figure 15 **b–d**). It is probable that its volume increases after new contingents are added to it, especially from the amygdala.

In times of Edinger and of Foix, the function and importance of the MFB were still unknown. After Panksepp (2015), it was illustrated and named for the first time by WE LeGros Clark in 1938.

#### 4.7.2 Dissection of the medial forebrain bundle

**FIGURE 15:**
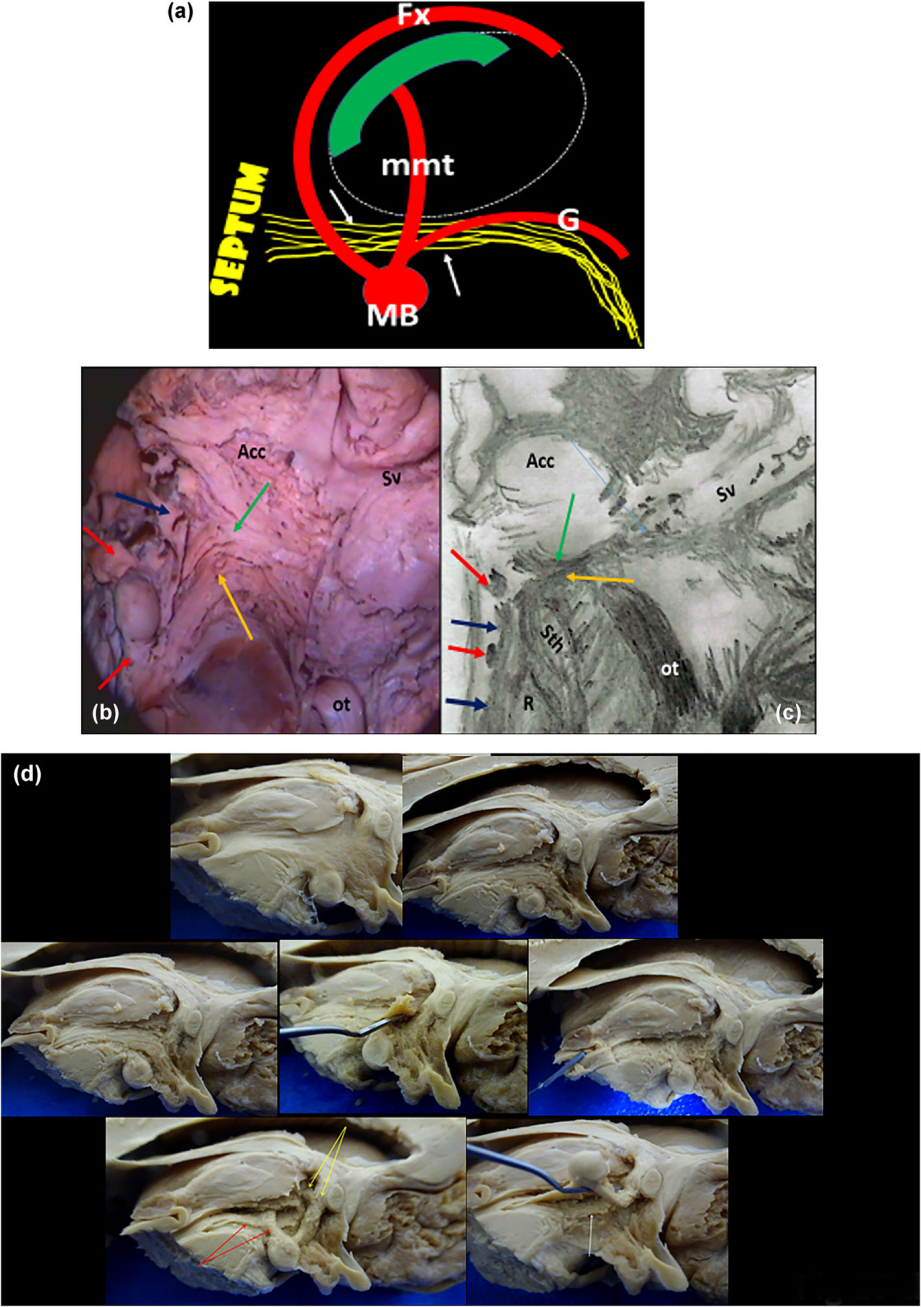
**(a) Outline** **of the medial forebrain bundle**. The figure shows the essential relation of laterality of the medial forebrain bundle (MFB) with respect to the fornico‐mammillothalamic‐tegmental formation. The representation of the bundle is inspired by a drawing of Edinger (1904) in the rabbit and of Nieuwenhuys et al. (1982) taken from Von Kramon et al. (1993) in man. The same position is assigned by both authors (**yellow band of fibers)** with respect to the fornix (**Fx**), the mammillothalamic tract (**mmt**), and Gudden's dorsal tegmental tract (**G**). **Green form,** anterior nucleus of thalamus; **little white arrows,** portion of the tract that can be exposed by dissection. For Nieuwenhuys, the MFB descends till medullary levels (Nieuwenhuys et al., 1982). **(c)** Horizontal section adapted from Foix & Nicolesco (1925) (drawn by us with pencil) at the inferior border of the zone shows a similar arrangement as our dissection (Figure 8), reproduced on the left. **B,** diagonal band cut at the origin of its vertical portion; **anterior red arrow,** anterior pillar of the fornix; **blue arrows,** the authors say “anteroposterior fibers”; **posterior red arrows,** mammillothalamic tract. **Sth,** subthalamic nucleus; **R,** red nucleus; **ot,** optic tract; **yellow arrows,** ansa lenticularis; **green arrows,** inferior thalamic peduncle; **Acc,** accumbens nucleus. The absence of structures at the center of the figure of Foix (between accumbens and inferior thalamic peduncle) is probably due to the concavity of the zone that prevents other presences at this low level of cut. **Sv,** sylvian fissure horizontal part. **(d) Progressive dissection of hypothalamus**. Extraction of the ependyma and the subependymal gray, exposing horizontal fibers and oblique fibers at the infundibulum. The fibers seem to form a fascicle that passes *medial* to the anterior pillar of the fornix. Reclining weakly the fibers, they become separated easily till the cut midbrain tegmentum. This is the *dorsal fascicle of Schütz* (Crosby et al., 1962). The fornix, the direct fornico thalamic (**yellow arrows**), mammillothalamic (**mmt**), and Gudden's dorsal tegmental tracts (**red arrows**) are exposed. The four elements are arranged initially in nearly the same sagittal plane. Laterally, between the fornix and the mmt appears another fascicle that is covered in part by the latter. Elevating the fornix–mmt–Gudden's tracts, the supposed MFB is visible (**white arrow)**. *Source*: (c) adapted from Foix and Nicolesco (1925).

### 4.8 Oblique coronal slices

We think these slices can help us to understand the intricate anatomy of the region. We have signalized each structure with a different color. A series of 1.5 mm separated oblique cuts with constant obliquity.

**FIGURE 16:**
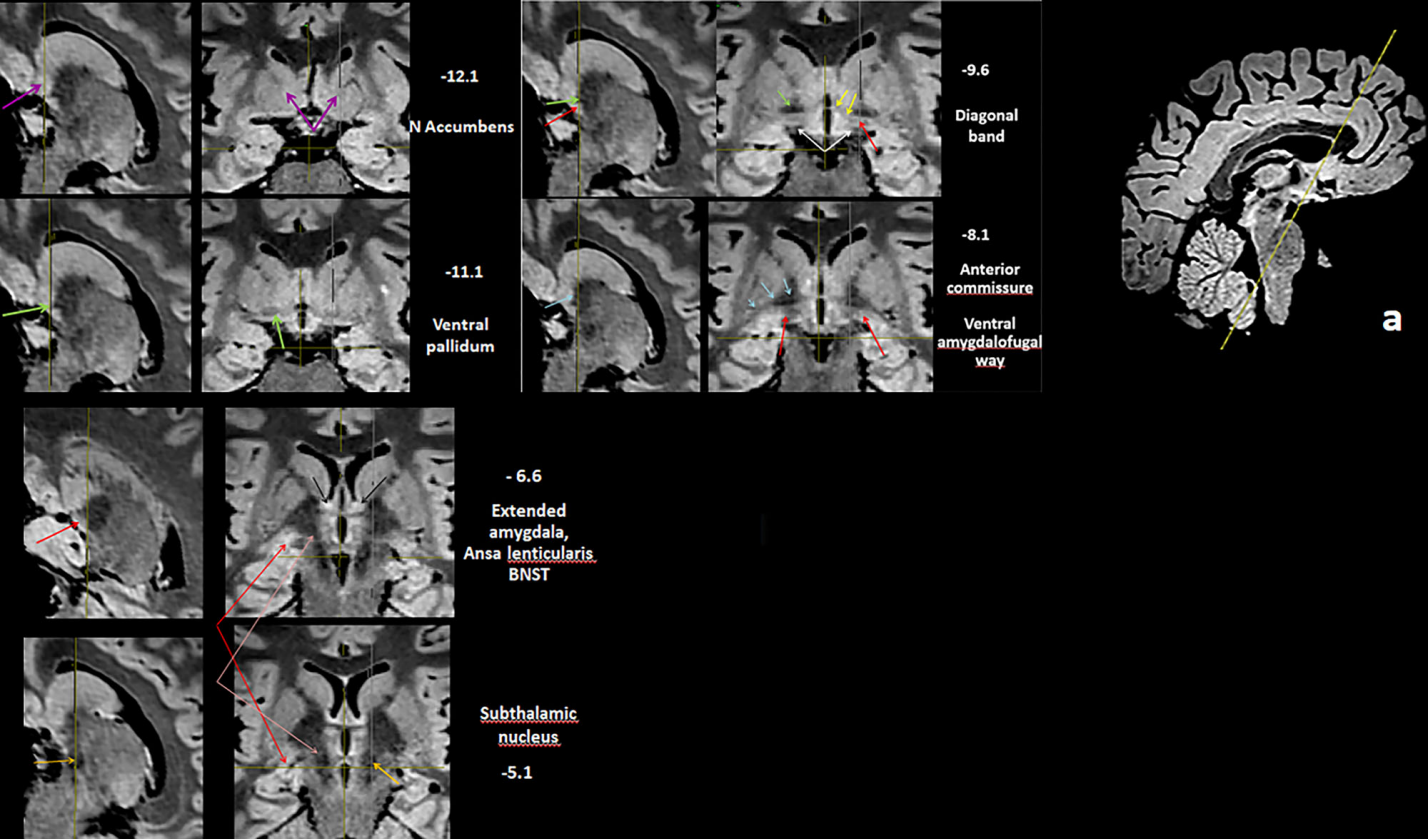
**(a)** Direction of cut. **(−12.1/−11.1) Purple arrows,** the nucleus accumbens has a higher signal than the head of the caudate (it has fewer fibers [Heimer & van Hoesen, 2006]) (see also Mavridis [Mavridis & Boviatsis, 2011]); **light green,** ventral pallidum. A huge striate vessel is visible in **12.1**. **(−9.6/−8.1): Yellow arrows,** diagonal band of Broca; **red arrows,** inferior thalamic peduncle (**IthP**). On the right side, the inferior thalamic peduncle makes contact with the bent of the ansa lenticularis (lowering of signal just at the point of the left red arrow). **White arrows,** optic tracts; **light blue arrows,** anterior commissure, posterior arm; **green arrows,** ventral pallidum. **(−6.6/−5.1); red arrows,** sublenticular extended amygdala; **pink arrows,** ansa lenticularis; **orange arrows,** anterior pole of subthalamic nucleus. Immediately medial to it the posterior poles of the mammillary bodies are to be seen. **Black arrows,** bed nucleus of the stria terminalis (BNST).

In these images, the ventral putamen reaches practically the surface of the APS, from which it is only separated by the islands of Calleja. The VP due to its low signal forms a clear posterior border for the accumbens in this sequence (Figure 16, **−12.1 and −9.6 sagittal cut**). There is a thick lateral striate vessel in the anterior part of the putamen with an unexpected anterior direction parallel to the cut. It can only be understood if one thinks that it comes from the APS, located posteriorly to the zone that it supplies.

Note in Figure 16 (**−9.6**) that the obliquity of the slice makes the dbB and IthP seem a continuous structure, because both come from the ventral amygdalofugal way. In −6.6/−5.1 the IthP is partially overlapped with the Al.

The BNST encircles the ac and continues with the hypothalamus without transition. In a thickness of 7 mm we have reviewed most of the principal anatomic structures of APS.

#### 4.8.1 Perforating vessels (PA) (Abbie, 1934; Andersen, 1958; Duret, 1874; Gillilan, 1968; Kaplan, 1965; Lazorthes, 1961; Rosner et al., 1984; Salamon & Huang, 1978; Salamon et al., 1966; Schlesinger, 1976; Westberg, 1966)

All the images are from our old series. The APS is one of the phylogenetically oldest parts of the telencephalon and also the hilum of the basal ganglia. The terminal carotid arteries bifurcate exactly under the APS and its terminal branches, the anterior cerebral and the middle cerebral or silvian arteries, and also the anterior choroidal artery, give rami that perforate the APS, to supply the head and body of the caudate, the lenticular nucleus and the highest portion of the internal capsule. Since 1874 (Duret's first description), these arteries received different names as lenticulocapsular, internal and external striate, pre‐ and postcommissural striate, but the currently preferred name is lenticulostriate given by Andersen (1958), a radiologist, in 1958 to the perforants of the silvian artery.

**FIGURE 17:**
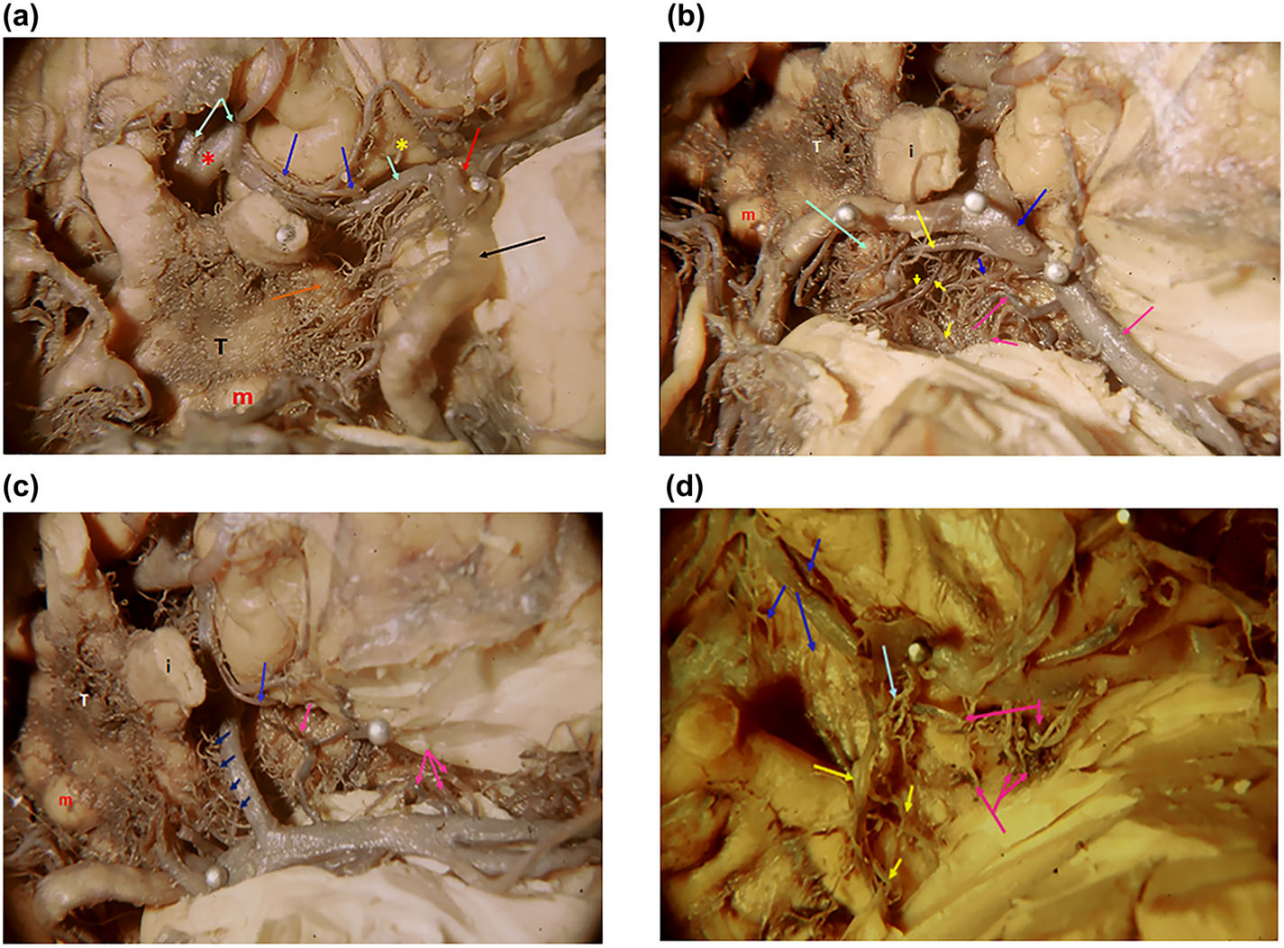
**(a)** Basal view of the brain at the level of the left anterior perforated substance (APS). Temporal pole partially resected. Left optic nerve slightly reclined downward. End of the internal carotid artery laterally displaced to expose A_1_. **Yellow star,** olfactory trigon; **m,** left mammillary body; **T,** tuber**. Red arrow,** end of the internal carotid artery; **light blue arrows,** anterior cerebral artery; A1 pointed with one arrow; A2 portions, an arrow each, united by a thick anterior communicating artery (**red asterisk**); **blue arrows,** Heubner's recurrent artery branching from the origin of A2 and perforating medially respect to the olfactory trigone, next to the entorhinal fissure. Other minor branches enter behind Heubner's artery, at the center of APS; **orange arrow,** left optic tract; **black arrow,** thick posterior communicating artery **(PcoA)**. **(b)** Same specimen rotated almost 90° to the right. Terminal internal carotid reclined medially (**blue arrow**); **blue little arrow,** perforators from the terminal internal carotid artery; **long yellow arrow,** anterior choroidal artery; **short yellow arrows,** its perforators to the posterior zone of APS. Their area of penetration is continuous with that of the rami of the bifurcating carotid artery; **Light blue arrow,** optic tract; **red arrows,** middle cerebral artery and its perforating rami; **shortest arrow,** branches that penetrate in the temporal portion of the entorhinal fissure. **T,** tuber; **i,** left optic nerve; **m,** mammillary body. **(c)** Supracarotid triangle, with limited resection of the posterior orbitofrontal convolutions. Temporal pole resected. **Long blue arrow,** Heubner's artery; **short arrows,** short perforators from A1 that penetrate slightly ahead of chiasma and optic tract; **lilac arrows,** perforating branches of the middle cerebral artery (lenticulostriate arteries). One of them (**lilac**) aims to the penetrating zone of Heubner's artery (**blue**). The lateral cluster penetrates in the most lateral part of the entorhinal fissure. Little rami not signalized penetrate in bare APS. **(d)** Another specimen (left APS). Temporal pole resected. Terminal internal carotid artery displaced forward. **Light blue arrow,** perforators from the bifurcation of the internal carotid penetrate at the center of the APS; **yellow arrows,** anterior choroidal artery and its perforating branches entering the posterior part of the APS. Observe the relation of the artery with the optic tract and how is the arrangement between them; **blue arrows,** A1 with its short perforators ahead of the chiasma and the optic tract. In the anteromedial APS, Heubner's artery is hidden because of the displacement of the vessels; **pink arrows**: Lenticulostriate arteries penetrating at the posterior entorhinal fissure or at the center of APS.

From PcoA originate multiple branches that do not perforate in the APS; they enter medially to the ot that becomes a natural barrier between two systems: telencephalic laterally, in the APS, diencephalic medially, at the tuber. Most of these arteries unite to form a vascular net in the surface of the tuber (Figure 17a). They are not but are assumed to be perforators. Only a few are truly perforating arteries (anterior thalamic perforators). Just at the bifurcation of the internal carotid originates a group of perforators that penetrate the posterior part of APS. Those vessels can also be branches of the anterior choroidal artery (AchoA). The AchoA crosses the ot almost longitudinally. Like with PcoA, the rami that penetrate laterally to the optic tract are APS perforators and aim at the pallidum and low internal capsule. Those issued after the crossing supply the thalamus and external geniculate body (Figure 17).

In Figure 17 **a–d**, we insisted in the place of penetration of the perforating vessels because, after Abbie (1934), the vessels that perforate in *the entorhinal fissure* are directed to the neostriatum (caudate putamen). Those that perforate in the bare APS aim to the paleostriatum (pallidum) and those that penetrate out of the fissure end in the other capsules or in the claustrum.

**FIGURE 18:**
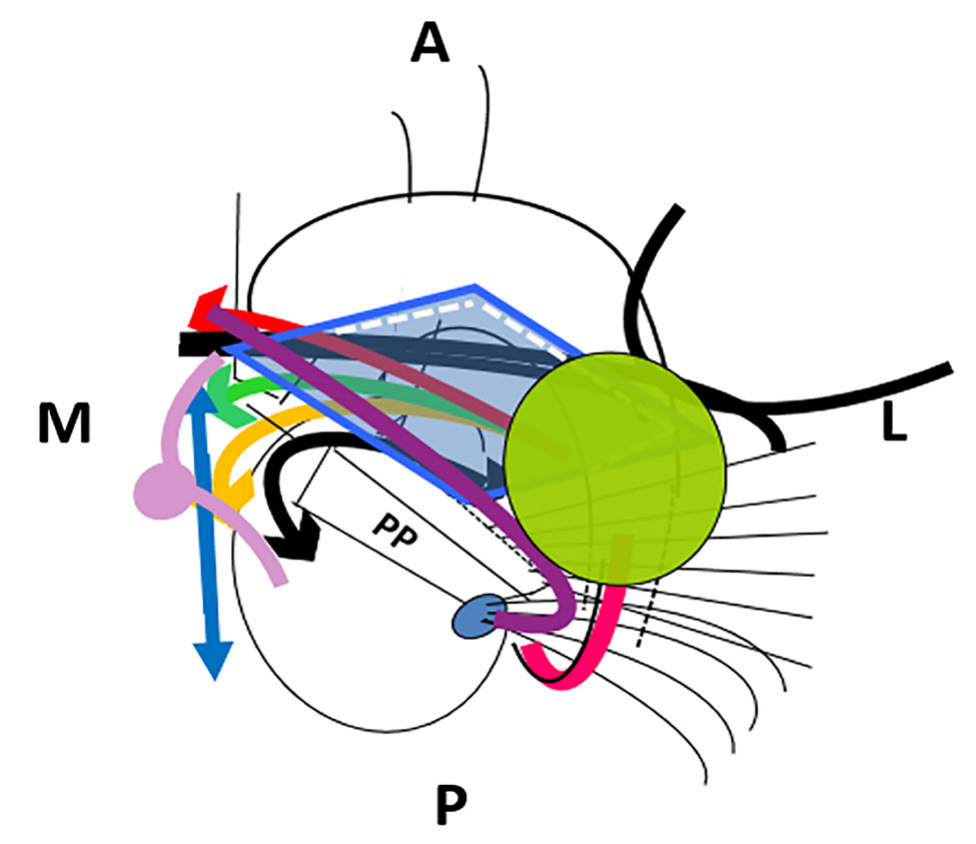
Initial outline (Figure 3c) showing the anterior perforated substance (APS) and the relation of the entorhinal fissure with the deep structures, here remarked with **the dashed white line** as eventual points of penetration.

In Figure 18, the transparency shows that the striatum is just over the anteromedial, the anterolateral and a part of the posterolateral portions of that fissure, making the observations of Abbie (1934) highly plausible.

The anterior angle corresponds to the zone of penetration of Heubner's artery and in deepness with the ALIC, the head of the caudate and accumbens, and the anterior portion of putamen.

The lateral angle corresponds to the penetration of the lateral lenticulostriate arteries that aim to the putamen, higher internal capsule, and body of the caudate.

Arteries that enter in the rest of APS would be pallidal.

#### 4.8.2 The short branches of A_1_

**FIGURE 19:**
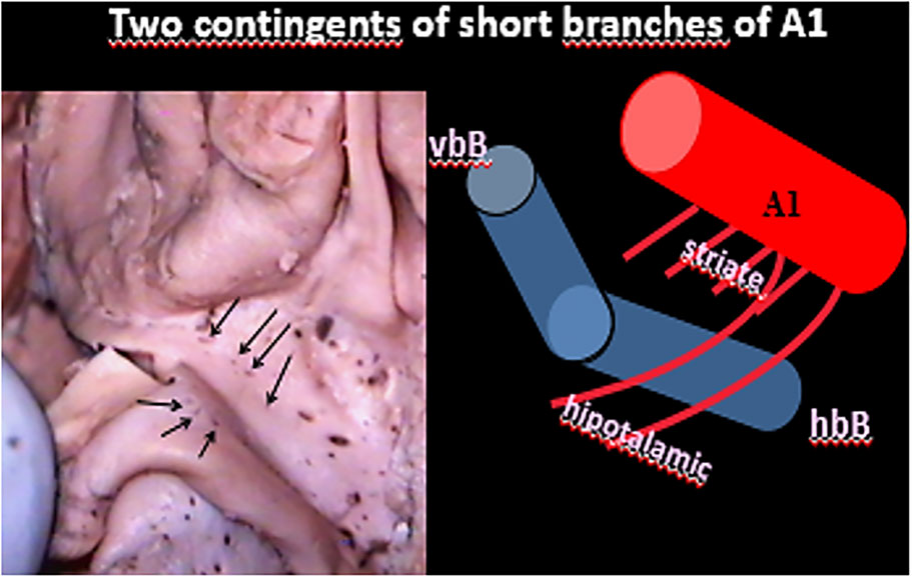
In our opinion, the posteromedial zone of the anterior perforated substance (APS) is perforated by short branches of the A_1_. This artery also supplies the suprachiasmatic zone.

The two contingents of short rami from A_1_ in Figure 19 are separated by the horizontal limb of the dbB. We propose to name the APS rami, as striate and those to the suprachiasmatic as hypothalamo‐chiasmatic.

#### 4.8.3 Intraparenchimatous course

**FIGURE 20:**
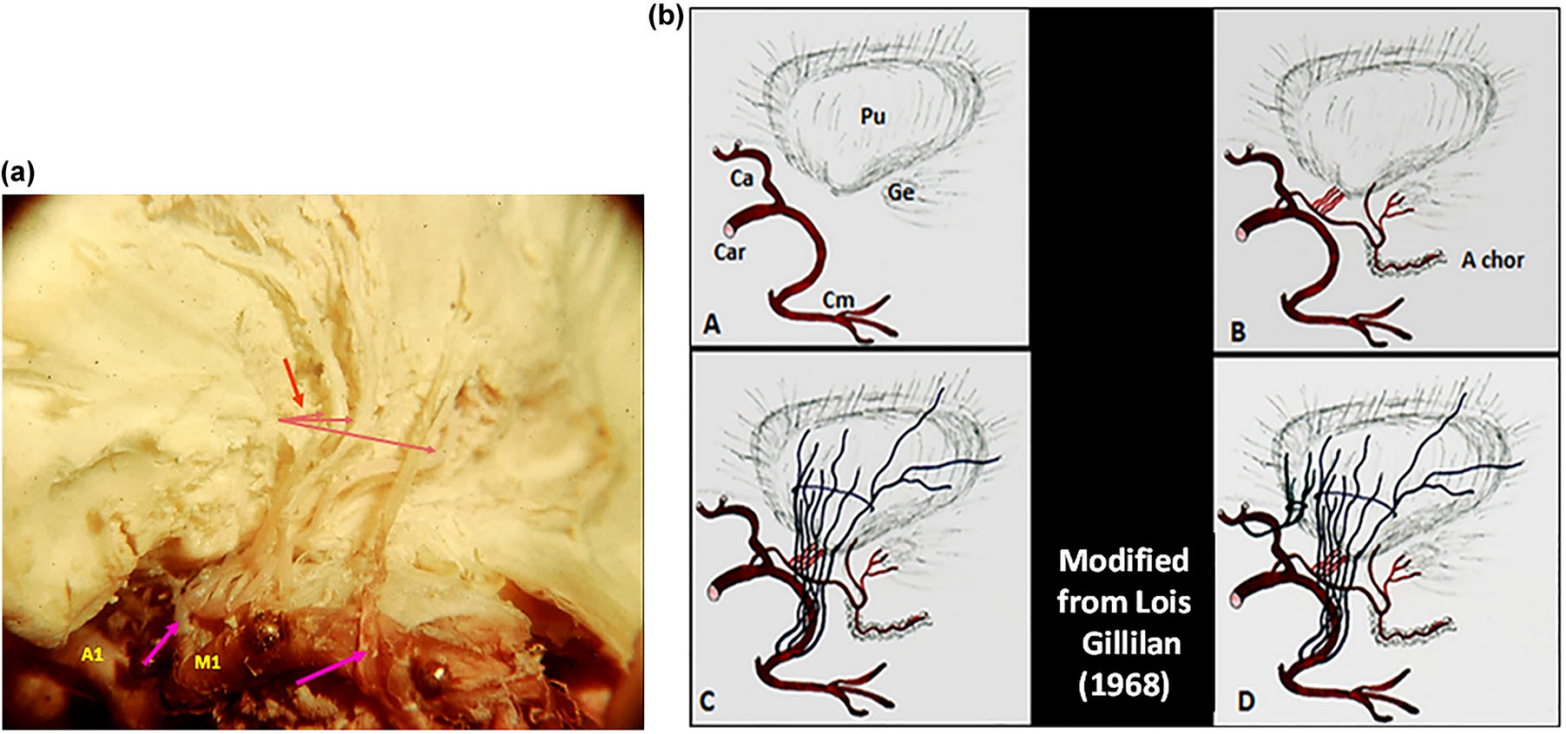
(a) Intraparenchimatous course of the lenticulostriate vessels in a left hemisphere injected with gelatin. Traditionally, of those vessels, the most internal are also the more anterior. (But see in [b] of this figure and also Figure 16a.) The pink arrow at left shows the non‐unusual origin of some perforators from a common trunk. The pink arrow at right shows a lateral ramus that has an openly recurrent trajectory before entering the anterior perforated substance (APS). **(b)** 3D outline of the course of the perforating branches by progressive planes. **A**: **Ca**r: internal carotid artery; **Ca** anterior cerebral artery; **Cm** middle cerebral artery; **A chor**: anterior choroidal artery; **Pu**: putamen; **Ge**: external geniculate body. **B**: Branches of the anterior choroidal artery. Through the APS enter rami for the pallidum, putamen, and internal capsule. Beyond APS, branches to the geniculate body, thalamus and choroid plexus. **C**: silvian rami (Lenticulostriate arteries). **D**: recurrent artery of Heubner. *Source*: Redesigned and modified from Gillilan (1968).

The image **in** Figure 20a shows the typical intraparenchymal bowing of those arteries that we had erroneously attributed initially to the passage of the anterior commissure. The very commissure is also curled as we have shown. Thus, it is due, more probably, to the development of forces during the growth of the brain especially the frontal and temporal lobes, in fetal age that produce progressive accompanying displacement of tissues and vessels.

The distribution of those vessels can be appreciated in Figure 20b.

**FIGURE 21:**
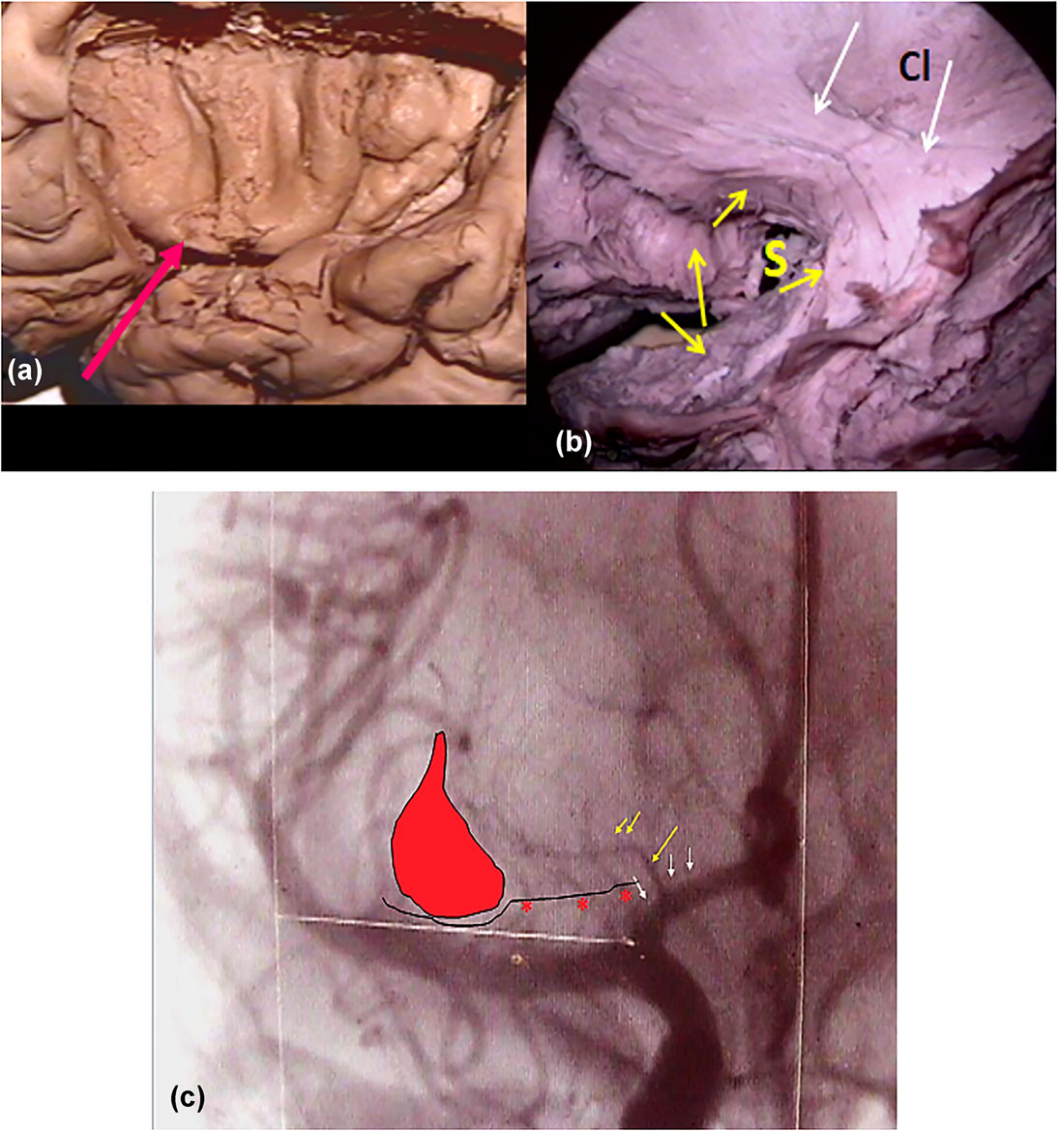
**(a)** Insula and dissection of the unciform fascicle and the frontotemporal extreme capsule fascicle in a left hemisphere—**left (a),** exposure of the insula by resection of the superior operculum of the sylvian fissure. The red arrow points to the threshold of the insula, the place below where pass the two fascicles shown in right. **(b) S,** horizontal portion of the sylvian fissure. **Cl,** claustrum; **white arrows,** inferior fronto occipital fascicle (**IFOF**), of which the frontotemporal extreme capsule fascicle is only visible; **yellow arrows,** orbital and lateral rami of the uncinate fascicle. The separation of both fascicles is somehow artificial. **(c) Angiographic detection of the anterior perforated substance (APS)**—**red asterisks,** signal the change of direction of perforant arteries. **Thin black line,** surface of APS and threshold of the insula; **red form,** represents the volume of the passing unciform and IFOF fascicles; **white arrows,** probably recurrent artery of Heubner; **yellow arrows,** anterior choroidal artery and little perforants from it.

The lenticulostriate arteries have a recurrent cisternal course that changes abruptly when they enter the APS (**Figures** 20 **and** 21c). Thus, they indicate the position of the surface of the APS in angiography. Lateral to the last lenticulostriate, pass the unciform fascicle and IFOF, between that artery and the first insular branch of the middle cerebral artery (Figure 21 **a–c**). The curved line that follows laterally the surface of APS signals the threshold of the insula/falciform fold.

## 5 DISCUSSION

### 5.1 The fibers

Both in 2002 and 2022, we could show that the dissection of the zone is affordable for any one that has a well‐formed idea of its anatomical relations and follows the directions and observations of the luminous paper of Klingler and Gloor (1960). Having better technical facilities makes sense but does not change the results too much. Good light and some magnification are enough. The rest is made by patience and meticulous work. It is true also that by the dissections, it was difficult for us to individualize and impossible to follow the amygdalo‐hypothalamic fibers.

We have observed that the different groups of fibers of the area differ remarkably in size between and intra‐subjects. (Figures 1c, 2 **a,** 9a,**b,** 11, 12b,**c**). We do not know of other researchers having the same findings. Being the zone one of preponderant psychic and cognitive significance, one could ask if differences of this kind would be at the base of interindividual differences, normal or pathologic, on the diverse capacities and reacting forms, in facing the daily eventualities of life. Caution must reign till we have the possibility to assess in vivo these differences with certainty and relate them to the psyche of the individual.

In the pathological realm, we have tried to derive the symptomatology of the syndrome of the anterior communicating artery, especially the memory troubles, from lesions of the APS and septo‐commissural area (Fontana, 2006). The clinical picture is similar to Korsakoff's syndrome, plus personality alterations that orient the analysis to the APS. Those symptoms have been attributed to lesions of the MFB, the accumbens, ventral striate and pallidum, “mediobasal areas,” and anterior cingulum. For us, there is a mechanism of multiple disconnections in these cases (Markowitsch, 2000): The hippocampus suffers a depression of its cholinergic innervation because of the lesion of the precomisural fornix in the septum. On the other hand, the mediodorsal nucleus of the thalamus can be partially deafferented through a lesion of the inferior thalamic peduncle in the APS, including also of the ALIC, supplied by Heubner's artery. Confabulation improves and disappears in variable whiles, but memory troubles remain, as well as the personality changes, sometimes positive, sometimes negative ones.

Surgically, the region has been proposed as a “gateway to the basal ganglia” (Rangel‐Castilla & Spetzler, 2014; Serra et al., 2019; Waldron & Lawton, 2009). We do not have clinical experience with the approach but looking at the intricacy of the little region and the “curtain” of perforant vessels, this does not seem the case, except for patients with a well‐delimited slow‐growing tumor that makes up its place by displacing the nervous and vascular structures, so opening a way to the surgeon. The approach proposed is through the supracarotid triangle of Kobayashi. The surgical entrance to the lesions is just ahead of the APS, through an incision in the posterior medial orbital convolution after ample opening of the silvian cistern and valley. The surgeon should keep up as close as possible to the surface of the tumor to avoid injury of the adjacent tissues and/or vessels. A pre‐, post‐, and late‐meticulous neurologic, neuropsychologic, and psychiatric assessment of those patients is imperative, although unilateral lesions could be better tolerated.

### 5.2 The inferior thalamic peduncle (IthP) and the ansa lenticularis (AL)

**FIGURE 22:**
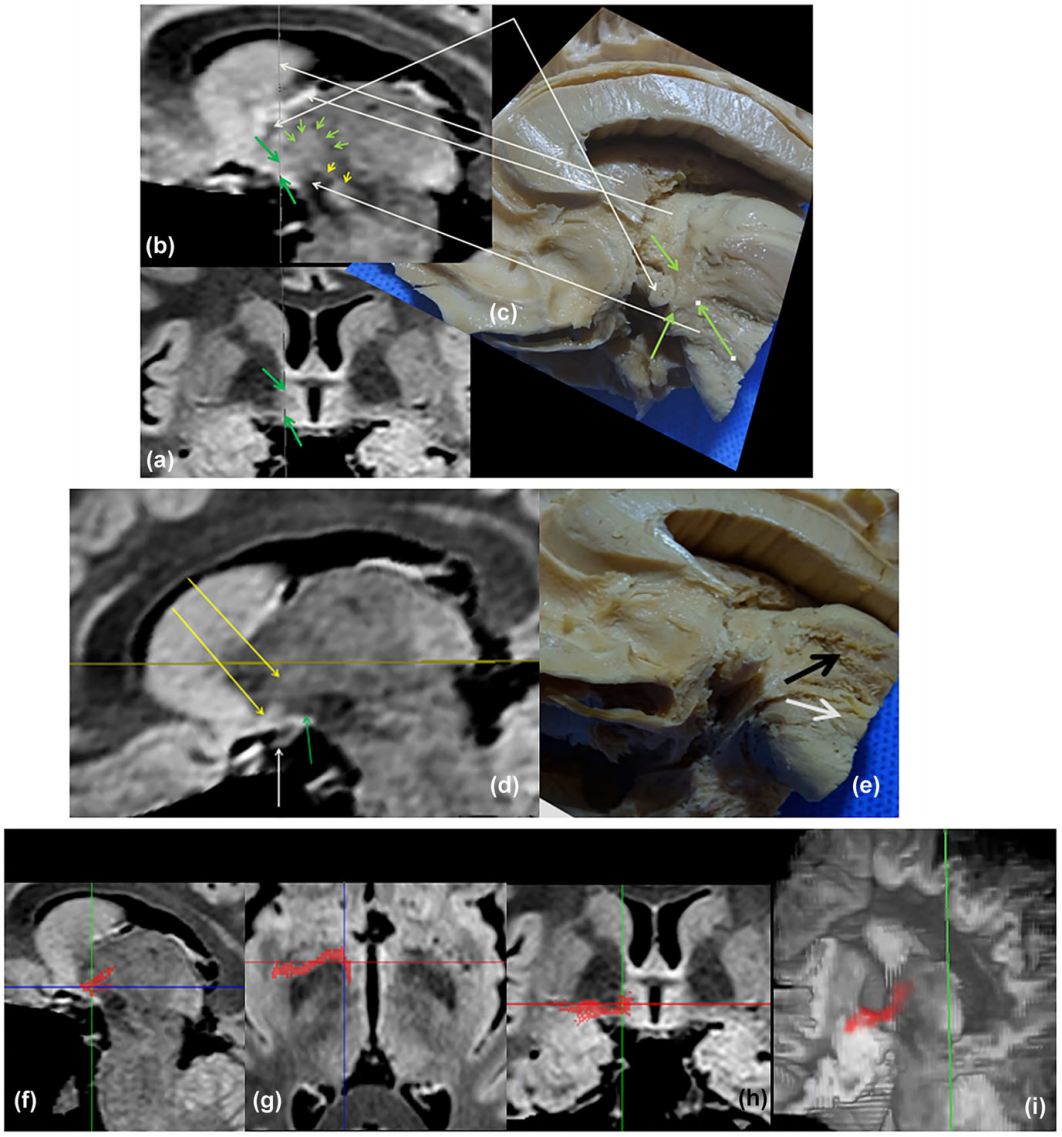
**(a)** Coronal MRI cut through the anterior commissure *y* = −5. Two **green arrows** mark the inferior thalamic peduncle where it seems to be changing its direction to parallel the sagittal plane, to reach its destination at the mediodorsal (MD) thalamic nucleus. **(b)** Sagittal cut that passes through the point of direction change at *x* = 7.5 mm. The **bigger green arrows** mark this same point as in **(a)**. The **little green arrows** encircle what seems to be the entrance of the peduncle in the thalamus. We are guided in part by the high signal of the structure compared with its surroundings. **White arrows** correlate structures from (c) in (b). From bottom to top—**ansa lenticularis** (contains the subthalamic nucleus) *with* subthalamic nucleus in **(b)**; **‐anterior commissure**; **‐dorsal bed nucleus of the stria terminalis** (in the dissection represented by a delicate strip of gray substance lying over the internal capsule. ‐**Head of the caudate nucleus**. Two yellow **arrowheads in (b)**: *zona incerta* (**Zi**). Below it, over the subthalamic nucleus, is well visible the lenticular fascicle. The thalamic fascicle is partially hidden by the arrows. All three structures seem interrupted by the ascent of the inferior thalamic peduncle ahead of them. **(d)** MRI sagittal cut 9.5 mm medial from the midline (in our opinion, the deepest zone of the inferior thalamic peduncle). **Yellow arrows,** vertical limb of the inferior thalamic peduncle; **green arrow;** ansa lenticularis. Both fascicles diverge just over the optic tract (**white arrow**). **(e)** Yet a known image shows the divergence of both fascicles. One superficial plane of cut fibers ends probably in midline nuclei. Both deep and superficial portions separate immediately after doing the ansa. In our opinion, its early fanlike division and the different planes of fibers could hinder the exact determination of its stereotactic coordinates of entrance in the mediodorsal thalamus. **(f)** We painted first the vertical arm of the inferior thalamic peduncle (**left**) and found that its inferior end coincides with the ansa its inferior or horizontal arm makes around the internal capsule just ahead and slightly inferiorly to the ansa lenticularis (**g**). The coronal view appears (in part) spontaneously once the other two are done (**h**). The result reconstructed in 3D shows the continuity of the peduncle viewed from medial and below (**i**). Unexpectedly, this painting had interesting consequences for our understanding of the zone, as will be seen.

Coming intimately in contact in their horizontal tracks, near the midline, both fascicles become sagittal and diverge widely to aim at their end points (Figure 12). Once in sagittal position, the direction of fibers in IthP is backward and upward. Instead, that of the ansa lenticularis and Forel's H zone is backward and downward (Figure 22 **a–e**). These and their diverse composition (IthP with probably low level of myelination and high cellular component, ansa lenticularis highly myelinated) help their clear differentiation in the MRI SPACE sequence. In the dissections, we observed that the inferior thalamic peduncle has a deep ending in the mediodorsal nucleus of the thalamus. It seems to be at 5–6 mm from the medial surface of the organ. In Figure 12 **right,** we have seen that the ending fascicle has several planes of fibers (at least two) the more superficial targeting the midline nuclei. In the deepest plan, we have found (one specimen only) that the fan is not so compact but formed by little divergent funiculi of different diameters. This finding could be coincident with the known paucity of amygdala fibers ending in magnocellular MD (Percheron, 2004) nucleus unhomogeneously in specific patches (Mai & Forutan, 2012) that could correspond to these funiculi we found.

The IthP we have dissected and followed in MRI (**Figures** 8, 11, 12, 22) is not the same fascicle Kamali et al. (2018) have described by tractography as amygdalo‐thalamic tract (ATT). We think tract investigation with tractography alone could be misleading. Although our anatomical support for the MR image of the IthP seems legitimate, we think that there could be a place for reasonable doubt about it. To reinforce our argument, we have tried to paint it in the brain register tool of the BioImage Suite program (Papademetris et al., 2006) and have had the results exposed in Figure 22e–i.

### 5.3 The medial forebrain bundle (MFB)

Although Panksepp (2015) credited LeGros Clark as the first to produce a schematic representation of the bundle in 1938, the first to describe it was SJM Ganser in 1882 (Nieuwenhuys et al., 1982), and we think that one of its first schematic representations was that of Edinger (1904) in a brain from a rabbit that we mention together with the outline of Nieuwenhuys in man. Both inspired our outline in Figure 15. Edinger named it *Riechstrahlung zum Mittelhirn*. Other authors, all German, also related the fascicle with the olfaction. The MFB is well developed in rodents, and the devoted species to its study is the rat.

Apparently, in 1924, Herrick was the first to give the bundle its definitive name (MFB) (Nieuwenhuys et al., 1982), although some authors think it was Edinger, to differentiate it from the internal capsule that is the *Lateral* Forebrain Bundle.

The tract became important after the experiments of Olds and Milner (1954), and the observation that in its trajectory there were points with high DBS stimulus reinforcing capacity (reward) and others in which the reinforcing diminishes (punishment). Therefore, it is important for learning, motivation, emotion, and memory.

In 1958 and 1961, Nauta (Nauta, 1961; Nauta, 1958; Nauta, 1962; Nauta & Domesik, 1982; Nauta & Kuypers, 1958) incorporated the MFB to the limbic system as its extension to the midbrain tegmentum together with other systems that have common hypothalamo‐mesencephalic continuity with it.

After the observation of Carlsson (Heimer et al., 2008; Yeragani et al., 2010) that the effects of reserpine were through depletion of dopamine in the CNS, a series of investigations by Swedish researchers found different loci in the brainstem that produce dopamine and also norepinephrine and serotonin. The ascendant ways of transport of them, all involved the MFB. Septal, BNST, lateral preoptic, lateral hypothalamic, posterior hypothalamic, habenular nuclei, and especially the centro‐medial amygdala and in slightly minor degree the lateral amygdala receive its dopamine through the MFB.

Both the dorsal corpus striatum and the ventral striopallidum receive their dopamine from the substantia nigra compacta (SNc) and from the ventral tegmental area (VTA), respectively, through the MFB or fibers in close association with it.

Moreover, several cortical areas receive dopaminergic innervation (Watabe‐Uchida et al., 2012) through the same system: medial frontal, medial prefrontal and dorsolateral prefrontal areas, anterior cingulate, medial fronto orbital, lateral orbital, medial prefrontal, and dorsolateral prefrontal areas.

It is important to remember that the different structures receive their dopaminergic innervation from gross topographically organized zones of the A8–A10 dopaminergic nuclei. In addition, this allows a reciprocal connection of the different telencephalic areas or nuclei with those medial midbrain tegmental areas. This could also theoretically explain the presence in the MFB of thicker myelinated fibers (Nieuwenhuys et al., 1982), along the thinner ascendant dopaminergic ones.

Thus, according to Björklund and Lindvall (1978), the whole meso‐telencephalic system is divided into a mesostriatal and a mesocortical system. The latter includes both the mesolimbic system (Ungerstedt, 1971) or meso‐allocortical and the meso‐isocortical of Björklund and Lindvall (1978). It is not clear after the descriptions of those authors *how* the ascending or descending fibers reach the cortex or the top of MFB, respectively. Recently, a superolateral division of the MFB (slMFB) has been proposed that from the prefrontal cortex would reach the bundle through the anterior limb of the internal capsule (Cho et al., 2013; Coenen et al., 2009, 2022; Hosp et al., 2019; Hurwitz et al., 2006; Panksepp, 2015). It has the drawback of having been traced only by tractography (Haber & Knutson, 2010; Haber et al., 2011, 2020; Haynes & Haber, 2013), in a zone like the hypothalamus that has uncountable fiber systems passing anteroposteriorly. Furthermore, a bundle of the MFB would be traveling through the *lateral* FB (internal capsule), which seems at least contradictory. If it truly exists, it should take another name. On the other hand, we must remember that the fibers should be continuous from cortex to VTA, as are continuous to SNc or subthalamic nucleus; Cho et al. (2013) also reconstructed by tractography the slMFB and described exactly its trajectory *through* the thalamus. They also believe to have found a new tract in the septum pellucidum (see Figure 10). The recognized effect of dopamine on reward has generated the importance of its study of MFB in psychiatry and drug abuse, and its interest for the treatment of some psychiatric refractory diseases by DBS.

Unexpectedly, we found in one of our first dissections in 2002, the short delicate strip located between the ascending fibers of the ansa peduncularis and the fornico‐mammillothalamic plane, coursing in anteroposterior direction through the lateral hypothalamus (Figure 8). Its anterior end, putatively in the septum, we could not find. The earned knowledge allowed us to recognize it easily in a recent dissection (Figure 15 **c**). In MRI, we could not identify it distinctly.

### 5.4 The gray substance

“The basal forebrain is a confluence of systems that are crucial to understanding the most important functions of the brain, including reward and punishment, learning and cognition, and feeding and reproduction” (Alheid, 2003).

“Due to the complex anatomy of the basal forebrain, the contribution of a specific anatomical system to a particular cognitive function has not been well understood”(Zaborszky et al., 2008). There seem to be two systems of the three that make up the gray substance in the room of the SI that can be identified by their MRI characteristics in the SPACE sequence. They are the ventral striopallidum and the EA. The latter, probably by its limbic character (Schneider & Vergesslich, 2007), gives the highest signal, especially at the BNST, so that one could speak about an “amygdaloid zone” of the SI. The VP, with abundant high myelinated fibers, sows the lowest signal. (Moreover, the iron deposition [Heimer et al., 2008] could have some influence in its low signal due to magnetic susceptibility, but our ES was too young for this factor to have influence.)

The VS emits an intermediate signal, a little bit higher (Mavridis & Boviatsis, 2011) than the rest of the striatum (contains fewer fibers [Heimer et al., 2008]).

**FIGURE 23:**
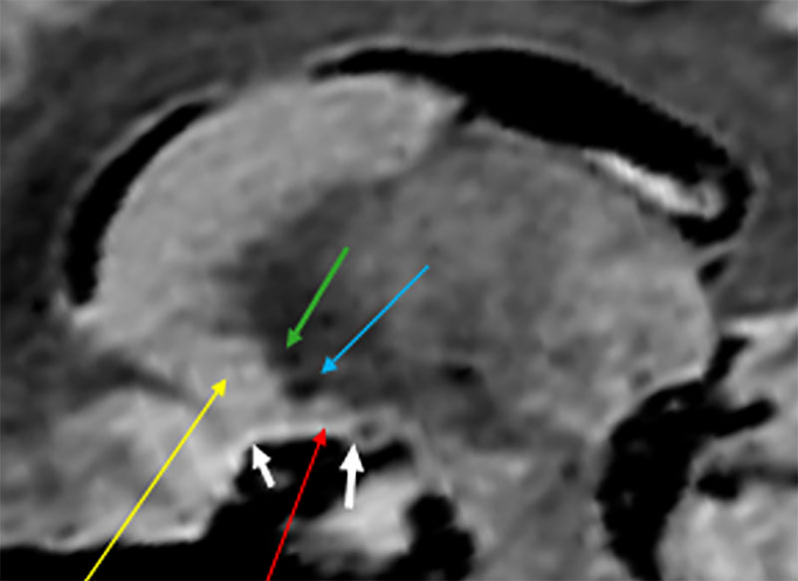
Sagittal cut at *x* = 13 mm. **Yellow arrow,** ventral striatum; **light blue arrow,** ventral pallidum; **red arrow,** extended amygdala (EA) with a point of low signal that represents the fibers of the inferior thalamic peduncle. It seems that the extended amygdala could actually extend over practically the whole surface of the anterior perforated substance (APS) as a thin lamella of fibers (deeper) and cells (shallow) of the rest of the ansa peduncularis, the more extended A–P, the more medially we are till *x* = 7.5 (see also Figures 3 and 8). **Green arrow,** anterior commissure; **white arrows,** limits of the APS at this level; **the anterior one,** entorhinal fissure; **the posterior one,** optic tract. The structures maintain this arrangement from *x* = 10 to *x* = 22 approximately. Note that neither the ventral pallidum, possibly, nor the ventral striatum reaches the surface of the APS.

So, we can say as a first approximation that from the SPACE MRI sequence, the room of the SI could be divided into three zones arranged anteroposteriorly. The first pertains to the VS. The second is to the VP. The third is to the EA (Figure 23).

#### 5.4.1 The basal ganglion of Meynert

**FIGURE 24:**
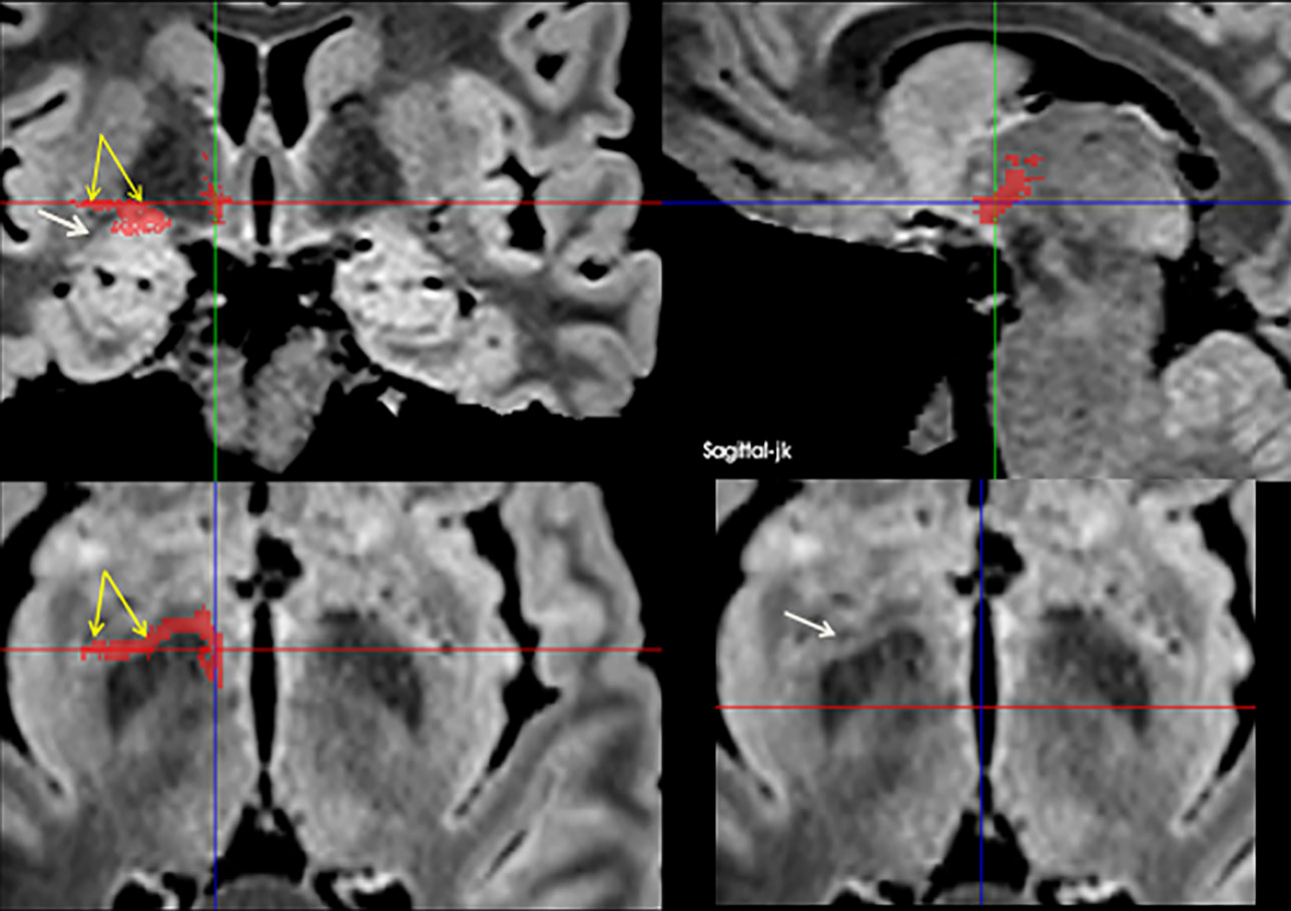
We painted the horizontal limb first in the axial cut. Looking at the corresponding coronal, we saw we were out of the inferior thalamic peduncle. The **yellow arrows** delimitate what is painted in excess, just below the pallidum and putamen, and at the top of the amygdala. **Right below**: the original axial cut. As seen in the coronal slice, we have probably painted the *sub putaminal nucleus* of Ayala and part of the nucleus of the ansa peduncularis of Meynert (or Ch4 al?). **White arrows,** anterior commissure. See also Figure 22h.

The ventral strio‐pallidum and the EA could be well discriminated with this sequence, but the BGM could have escaped our recognition because we do not have MRI elements but only some anatomical clues to identify parts of it. Halliday et al. (1993) have the same argument. We adopted it. The similarity of signal could induce to mistake a structure for another **(see** Figure 24). Here we found the first clue. We mean the region in which two parts of the basal ganglion seem to fuse.

We repeated the experience of painting the IthP but now beginning with its horizontal limb **(see** Figure 24 and compare with Figure 22). By painting, we had made a mistake. We painted excessively lateral, a zone that has the same signal intensity as the inferior thalamic peduncle but *that does not belong to it*. Beginning here and trying to find clusters of the BGM, we have searched for those related to the ansa peduncularis (intermediate Ch4 after Mesulam).

**FIGURE 25:**
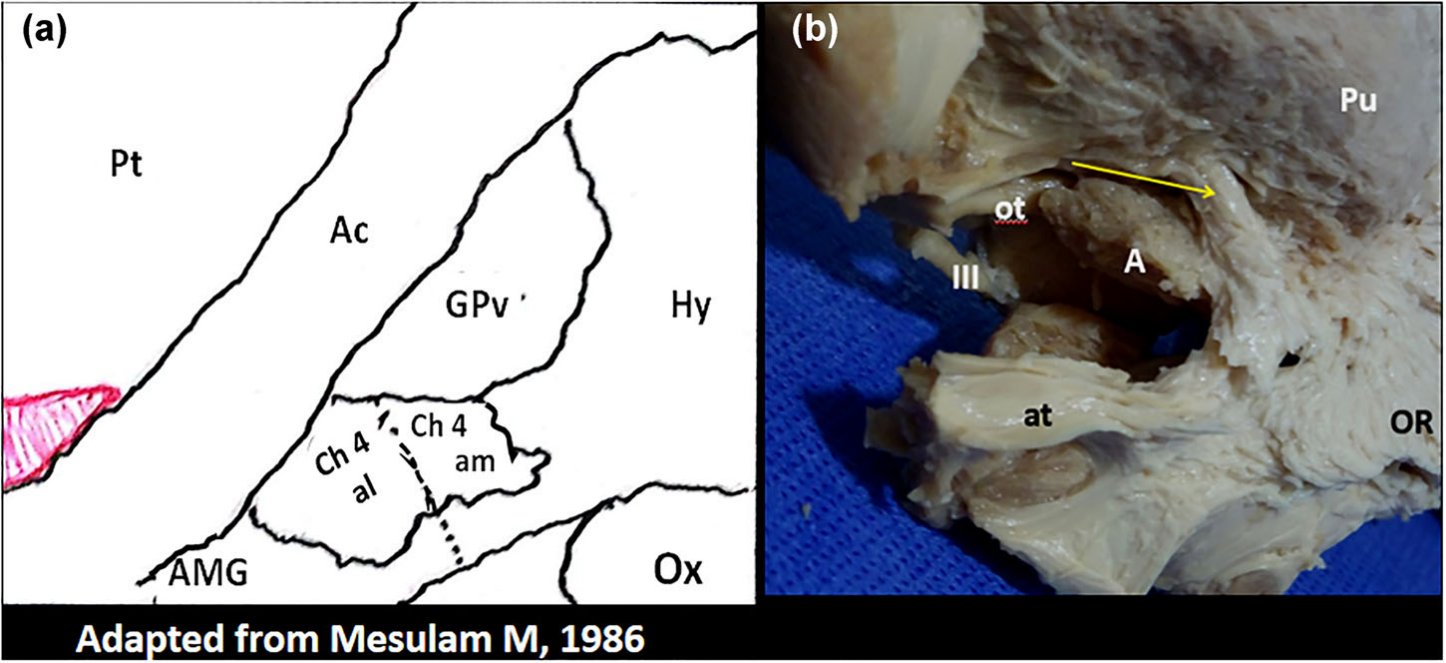
**(a)** Outline of a histological cut of a brain of *Macaca mulatta*; we added with the **red form**, the approximate position of the subputaminal nucleus of Ayala. The anterior commissure passes between both nuclei. Observe the apparent continuity of the Ch4 al and am with the amygdala (AMG). By painting the inferior thalamic peduncle, we have erroneously painted this zone that does not belong to the extended amygdala‐ventral amygdalofugal way, but to the basal nucleus. It is continuous with a hyper intense signal region, lateral to the optic tract, when it encounters the cerebral peduncle‐internal capsule border well described by Zaborszky et al. (2008) **(asterisk in** Figure 3 **c)** (see **Figures** 26 **and** 27) continuous with the amygdala in the SPACE sequence, because both tissues have a similar signal. **GPv,** pallidus ventralis; **Hy,** hypothalamus; **Ox,** optic chiasm. **(b)** Relations of the above figure in the human brain. Dissection made from lateral (left hemisphere). Uncinate and IFOF fascicles and external capsule resected. We can see the putamen (**Pu**), the anterior commissure emerging below it (**yellow arrow**) and over the amygdala (**a**); **ot,** optic tract; **III,** third nerve; **at,** amygdalo temporal tract; **OR,** Meyer's loop. *Source*: Adapted from Mesulam et al. (1986).

Logic indicates that they should be proximal (lateral), a place in which the fibers of the AP travel closely (**see** **Figures** 9 **b and** 12). The anterior commissure crosses the pallidum through Gratiolet's canal to emerge over the amygdala and below the putamen and follows between them till the external capsule. In this final track, the commissure is surrounded by Ayala's subputaminal nucleus (Figure 25 **a,b)** externally and dorsally, separating it from the putamen and by the nucleus of the ansa peduncularis of Meynert medially and ventrally separating it from the amygdala. After Ayala, the commissure would be *almost* completely surrounded by these nuclei. Immediately behind them, commences the intermediate part of the ganglion that corresponds to the ansa peduncularis (Ch4 id and v after Mesulam et al., 1983, 1984, 1986). Possibly, Ayala's nucleus *is not cholinergic* because it is not registered in Mesulam's and more recent investigations (but see Simic et al. (1999)).

**FIGURE 26:**
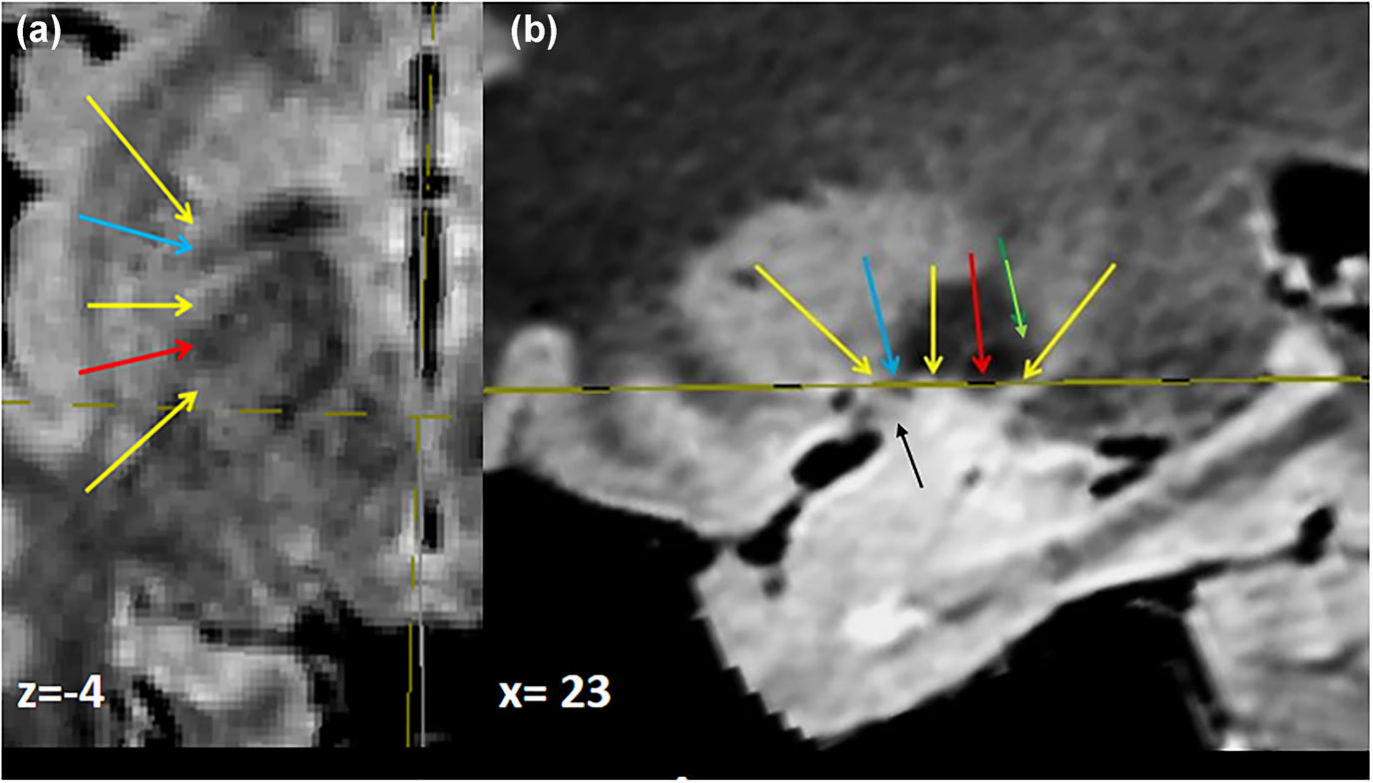
**(a) Light blue arrow,** anterior commissure surrounded by basal nucleus as described in text; **yellow arrows,** clusters we would interpret as pertaining to the basal ganglion; **red arrow,** ansa lenticularis (AL) exceptionally well seen in its two components (Lopes‐Alho et al., 2019) **(see** Figure 3). The **middle yellow arrow** points to the inferior thalamic peduncle. Note the low volume of its fiber component at its origin with a “target‐like” form. **(b)** Sagittal cut through the same place (*x* = −23). The clusters are in relation with the highest portion of the amygdala. Arrows have the same meaning. **Last (right) arrow,** posterior portion of Ch4 that seems to ascend and progressively decrease its signal in the internal capsule. A tenuous prolongation seems to enter the medullary lamina of the pallidum (**green arrow**). The lenticular ansa protrudes from below the globus pallidus (**red arrow**). The **middle yellow arrow**, ahead of the ansa lenticularis, corresponds to the inferior thalamic peduncle surrounded by dorsal and ventral intermediate Ch4. **Black arrow**: a plane of fibers that originate in the amygdala and travel in the surface of the anterior perforated substance (APS) medial and forwards and that forms part of the ventral amygdalofugal way (see following figure) and the diagonal band. A vessel enters in front of it.

Looking for clusters of the basal ganglion, beginning with those just mentioned and maintaining Ayala's idea of “proximality” (laterality), we searched the basal region with cuts in the CA–CP plane. At *z* = −3.5, −4 between *y* = −0.5 to 14 and we found a line of possible candidates. (We will see that it reaches ahead till *y* = −2.) The arrows in Figure 26 **a,b** are just in this line.

#### 5.4.2 The ventral amygdalofugal way (VafW)

**FIGURE 27:**
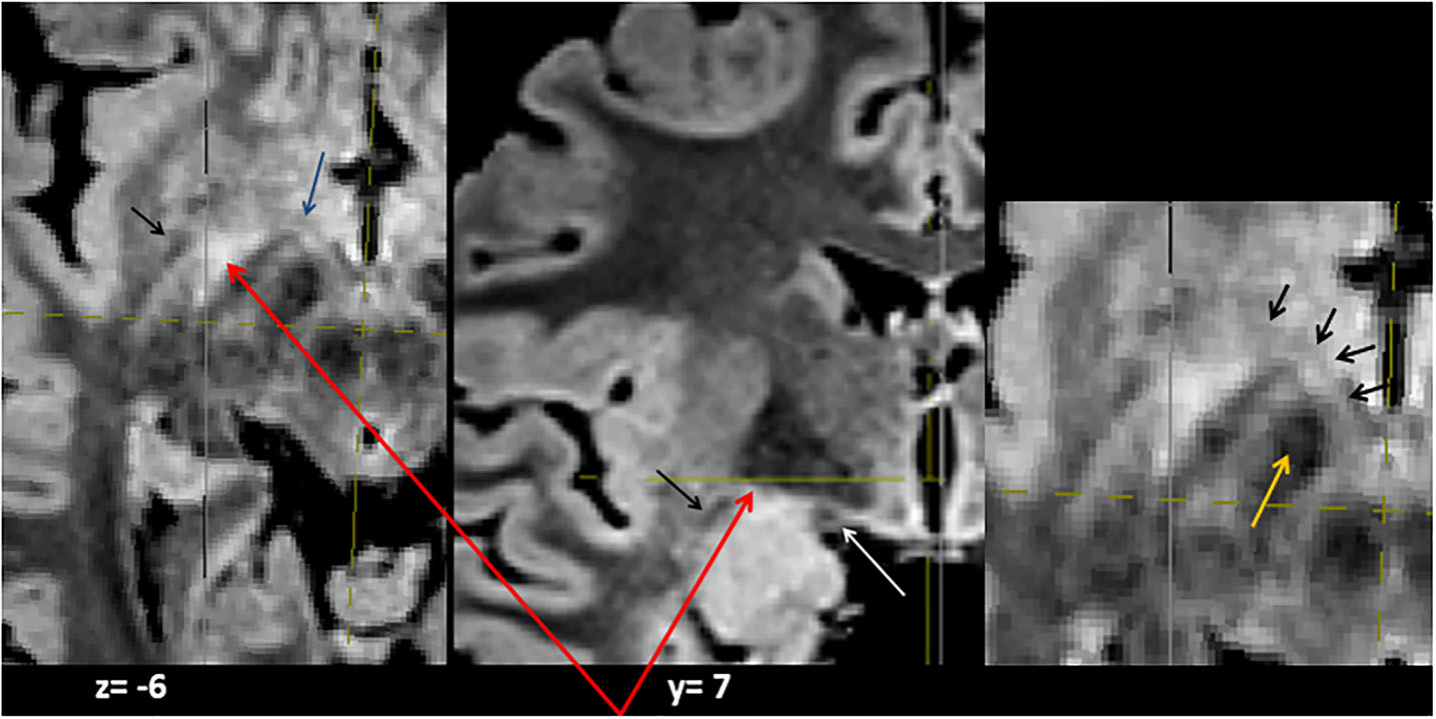
**Ventral amygdalofugal way**. Triangular area pertaining to the basal ganglion of Meynert (Zaborszky et al., 2008) **(see** Figure 3 **right)**. Cut parallel and 2 mm below (**c)** of the previous figure. **Black arrows,** anterior commissure; **white arrow,** optic tract; **red arrows** unite the corresponding zones in both slices. There are also the internal capsule, red and subthalamic nuclei, and the arrival of the post‐commissural fornix at the mammillary bodies; **blue arrow,** the suspected ventral amygdalofugal way. In the magnified image, it can be appreciated the relations of this “ansa,” as said, with the anterior border of the internal capsule and the subthalamic nucleus (**orange arrow**).

In the above image (Figure 27), we can see the triangular zone of Zaborszki et al. limited ahead by a slender fascicle from the APS that seems to enter the ventral tegmental area after a brief “ansa” around the anterior border of the internal capsule in relation with the subthalamic nucleus and finally, seems to end in the VTA, in front of the red nucleus. The fascicle must be part of the ansa peduncularis or ventral amygdalofugal way and incorporates to (or comes from) the MFB. The previous figure is of especial anatomical richness. We will try to disentangle the exposed structures that constitute the VafW.

**FIGURE 28:**
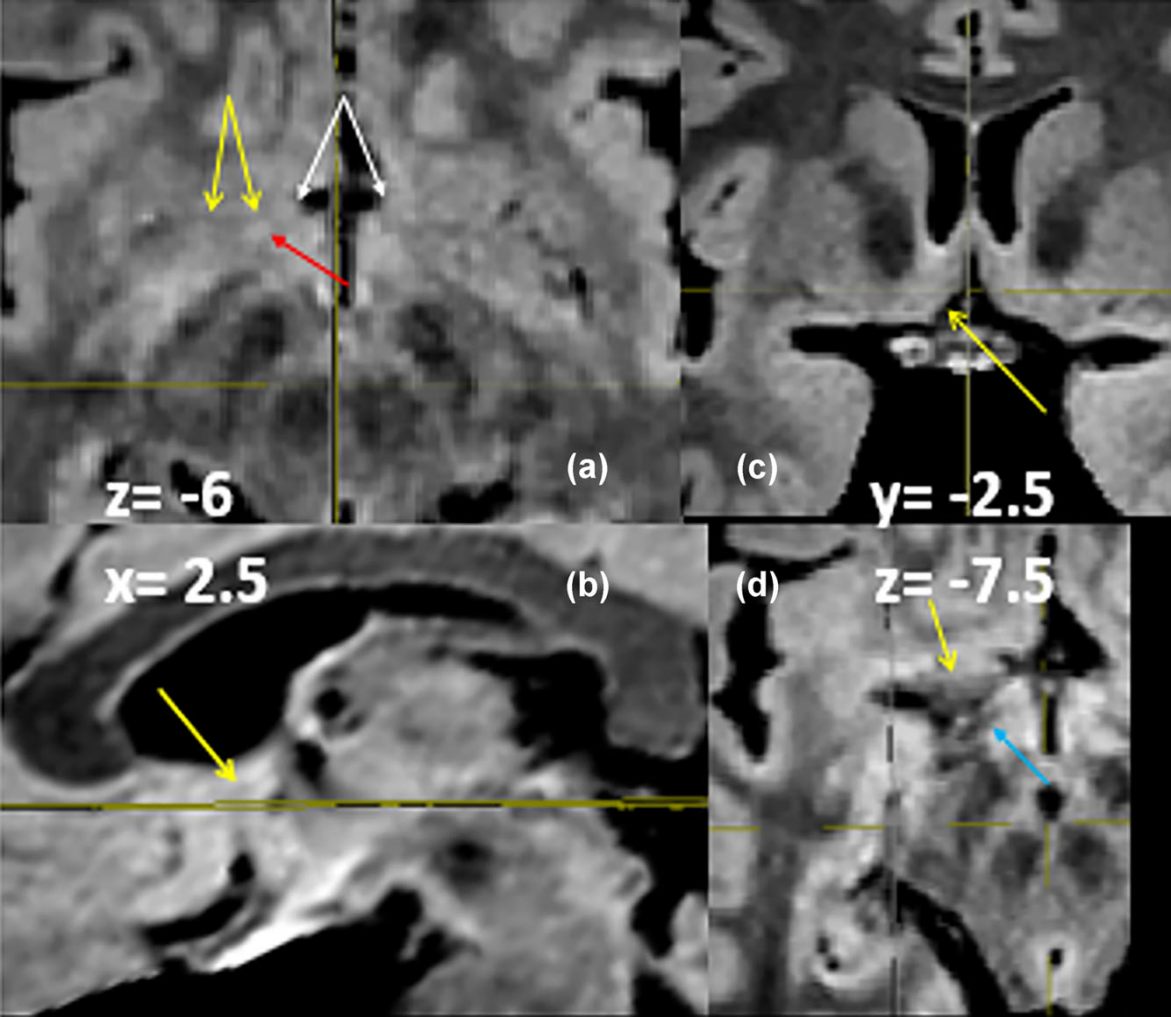
**(a) White arrows,** sulcus in which depth lays the vertical limb of the dbB. The posterior wall of this sulcus is the preoptic region (**a**). The lamina terminalis that separates the vertical diagonal band from preoptic region is not visible in (a). The deepest part of the sulcus is at 2.5 mm from the midline (**c)**. **Yellow arrows,** horizontal limb of the diagonal band in **(a)**; **red arrow,** inferior thalamic peduncle that changes direction to ascend to the thalamus. One millimeter below (**d**), the horizontal limb of Broca's band passes skimming the surface of the anterior perforated substance (APS). **Light blue arrow,** optic tract. In **(b)** and **(c),** one can see the “traction” the band exerts on the infero‐medial surface of the brain: the widest place of the basal interhemispheric fissure.

Figuratively, the two parts of the dbB act as a girth that constrains the tissues at the level of septum verum. Thus, the APS is elevated, and the medial surface forms the described deep sulcus (Figure 28a–d), for us “the widest portion of the ventral interhemispheric fissure” **(see also** Figure 2 **d)**.

**FIGURE 29:**
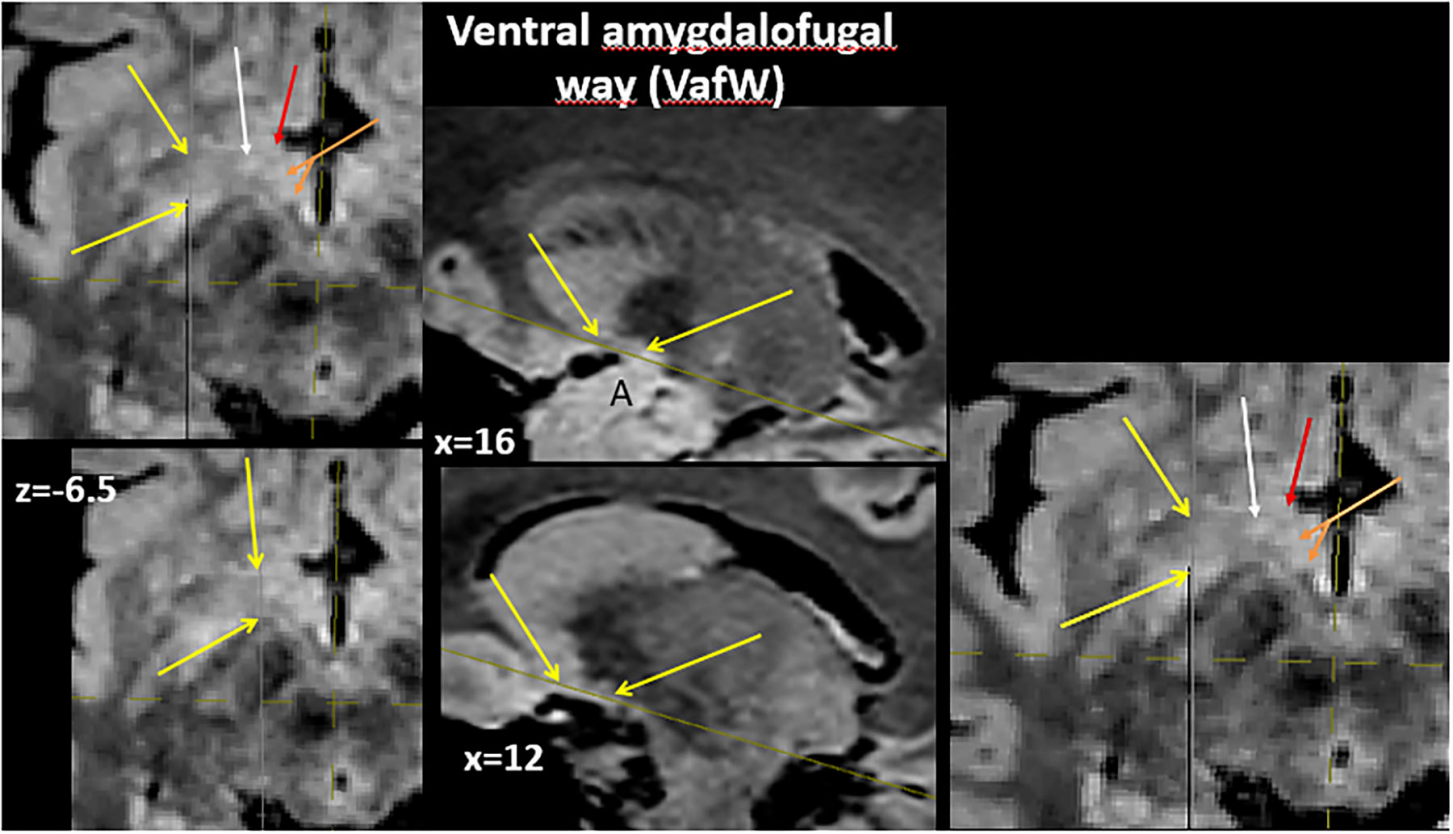
A panoramic glance allows visualization of the VafW at level *z* = −6 cut at two sagittal levels to show its continuity (**yellow arrows**). **(A)** amygdala. The path contains posteriorly, the descending band to the VTA, and anteriorly fibers that ascend or descend to the hypothalamus (**orange arrows**), the diagonal band (**red**), and the inferior thalamic peduncle (**white**) all seeming big higher signal ovals that distinguish over the lower signal fiber path. Better visible on the enlarged side image. See also **Figures** 3 **and** 8. In the cut *x* = 16, the VafW seems to be continuous forwards with the fibers of the external capsule.

In Figure 29, the ventral amygdalofugal way can be well seen in a horizontal cut at *z* = −6.5 mm.

**FIGURE 30:**
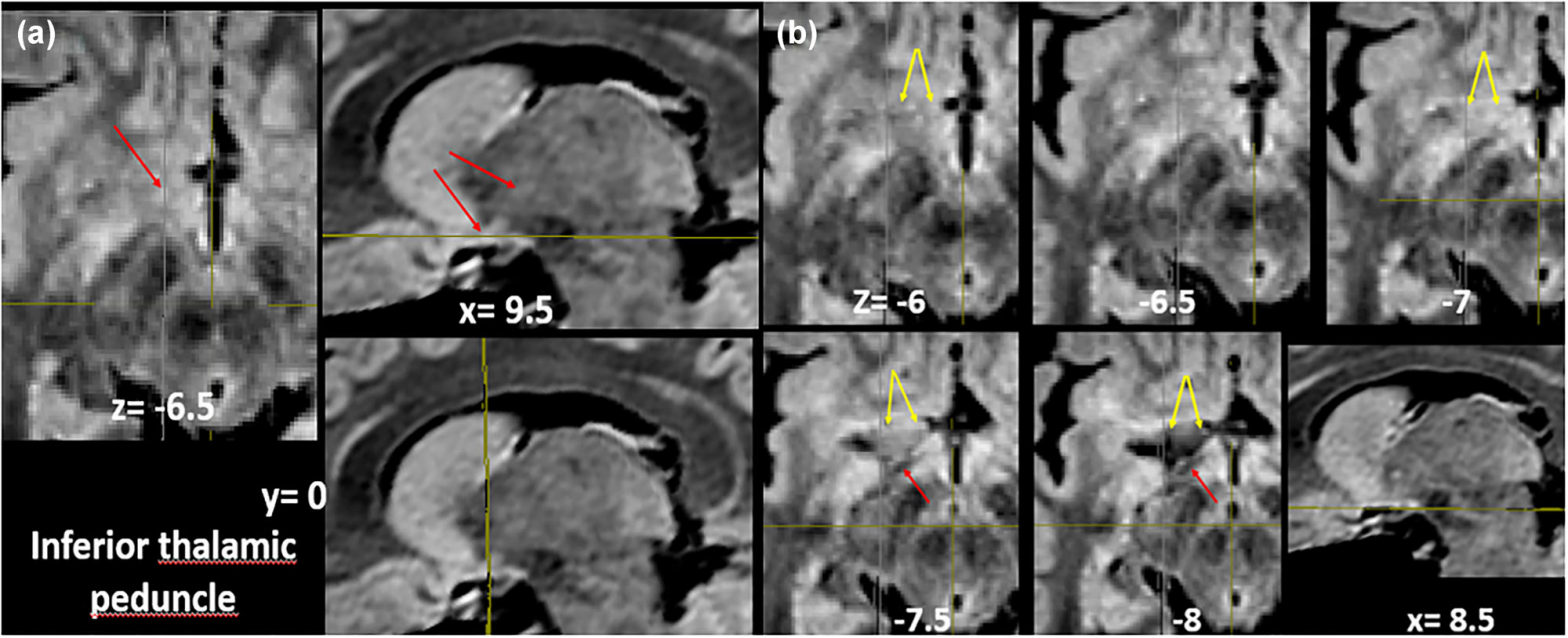
**(a) Inferior thalamic peduncle**. The lowest and more medial point of the bend the inferior thalamic peduncle makes to reach the mediodorsal nucleus of the thalamus (**red arrows**). **(b)** Ventral amygdalofugal way. A run of 0.5 mm slices parallel to the CA–CP plane from a level immediately below the ventral pallidum, till the surface of the anterior perforated substance (APS). **The yellow arrows** point to the diagonal band of Broca. Observe that in the two last images a portion of the diagonal band composed of fibers and another of grey substance are visible (*z* = −7.5). **Red arrows,** optic tract.

In Figure 30, we made a sagittal cut passing through the “oval” supposedly being the point of the bend of the IthP. Effectively it is, as seen in the sagittal cut. The deepest ascending portion of the IthP is visible. Compare with Figure 22b. In **Figure** 29**e**, the horizontal dbB seems to be cut longitudinally and shows a “two bands aspect,” one of high signal forward and another low signal behind.

#### 5.4.3 Ch4 al and ai portions of the BGM

**FIGURE 31:**
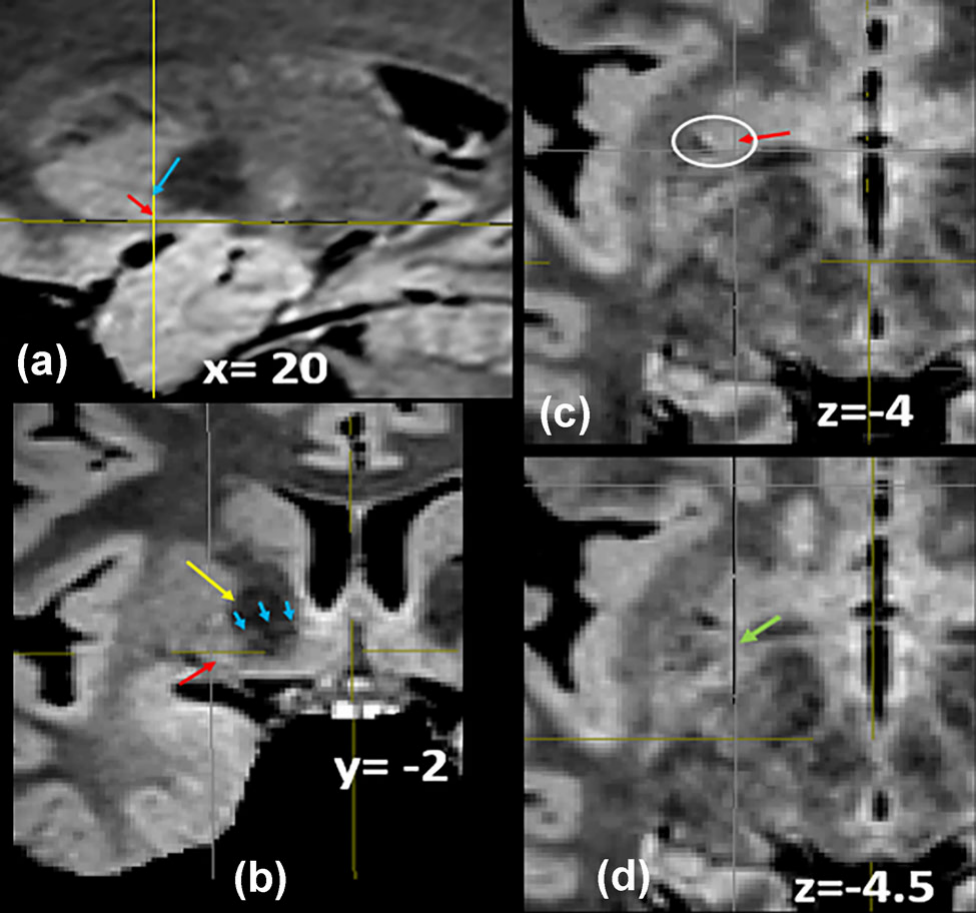
**(a) Sagittal section,** level of CA–CP planes **(c)** and **(d)** at *z* = −4 and −4.5 passing through the gray between ventral pallidum and ventral amygdalofugal way, with **(b)** coronal plane tangential to the anterior bow of the ca (**light blue arrows**) (*y* = −2). **Yellow arrow,** lateral medullary lamina of the lenticular nucleus. Part **(c)** is the same slice of Figure 26, where we recognized the compact posterior and intermediate portions of the basal ganglion of Meynert. **Red arrows in the three images,** probably Ch4 al; **white circle,** relation of this tissue with the lateral lenticulostriate vessels. **(d)** Zone just below the lamina externa that could correspond to Mesulam's *anterior intermediate portion* of the basal ganglion of Meynert (Ch4 ai) (**green arrow**). See text below.

The VafW is accompanied by a gray substance: a mixture of cells of the EA plus cells of the BGM. The last is perhaps more numerous in their place of preference: below the pallidum.

In the inferior portion of the external medullary lamina of the LN, the **ac** arrives at the inferior face of the putamen (Figure 31) being in part surrounded by gray matter that distinguishes clearly from the pallidum by its higher signal in this sequence (compare with Figure 5c). We think it belongs to the BGM that forms an incomplete layer around the ac as is described (Halliday et al., 1993; Hedreen et al., 1984; Tenenholz‐Greenberg & Heinsen, 2007; Zaborszky et al., 2008). In this zone of the lamina, there are other clusters of high signal visible (Figure 31b). There is a zone just below the lamina that could correspond to the *anterior intermediate portion* of the BGM (Ch4 ai) recognized by Mesulam and Ch (1988) only in the human. Lateral lenticulostriate vessels traverse the zone (Figure 17c) for some authors an orienting landmark for this portion of the BGM (Hedreen et al., 1984; Tenenholz‐Greenberg & Heinsen, 2007; Zaborszky et al., 2008).

#### 5.4.4 General discussion of BGM

We can agree that there seems too much knowledge extracted from these very images.

Surprisingly (for us), the nucleus basalis appeared in continuity over the amygdala. We have learned it is true, but only in the human and humanoid primates. The phylogenetic studies of Brockhaus (1942) in microsmatics showed that originally, the compact lateral portion of the nucleus was *behind* the lenticular nucleus and extensions of it in the internal medullary lamina were the rule. In humans (Brockhaus, 1942), the growth of the lenticular nucleus and a displacement of the BGM laterally and anteriorly made possible its appearance *below* the lenticular nucleus (preferentially the pallidum) and *over* the amygdala. The extension to the medullary laminae passed from the internal to a predilection for the external lamina. From both of these positions, the BGM could reach the internal capsule.

In their atlas of monoclonal choline acetyltransferase and acetylcholinesterase histochemistry, Mesulam et al. (1984) found the distribution of positive cells in the macaque has a homogeneous somehow diffuse pattern at least in the SI, except in the proper zones of the ganglion (compact vs. diffuse zones of Brockhaus (1942)). On the other hand, in the sequence here studied, both amygdala and ganglion seem to have a similar high signal. We could say that with maybe, some exceptions like the BNST, the structures studied are not unique but composed of a heterogeneous substance made of specific fibers but of cells of different systems. The mix has a high signal in the sequence here applied and possibly in others.

#### 5.4.5 The topography of the BGM in the substantia innominata

After our measurement, the compact basal ganglion extends in the mediolateral plan between *x* = ±18 and *x* = ±24.5. In the anteroposterior plan from *y* = −2 to *y* = 14 and in the vertical plan from *z* = −3 to *z* = −6 mm.

The arrangement is different for the Ch3 and the anteromedial Ch4 that were more difficult to identify for us with this sequence. The Ch3 is a transversal band of gray that accompanies the fibers of the dbB. We found it at *y* = −3 and *z* = −7.5, from *x* = 18 to *x* = 0.6. For the Ch4 am, we do not have coordinates to offer.

So viewed, the basal nucleus plus the nuclei of the horizontal dbB adopt a form of an inverted letter L with the long limb directed backward and lateral, and the two short limbs directed to the midline. The structure seems to be continuous but with strictures and expansions (we do not consider here Ch1 and 2 treated in **Figures** 2 **and** 5).

#### 5.4.6 MRI and the substantia innominata

Notable achievements have been made recently for the MRI visualization of the structures of the basal brain in normal material ex vivo (Hoch et al., 2019). We do not have knowledge of previous authors reporting differences of tissue signals in the region of the SI in MRI in vivo or anatomical analysis that could have eventual importance for the recognition of grey structures except may be for the low signal of the pallidum. Till now, the MRI studies of the area have concentrated in the measures of volume or height of the grey substance to detect diminutions that could point to some degenerative diseases of the brain, like AD, PD, LBD, Korsakow's syndrome, and other conditions (Arendt et al., 1983; Fernandez‐Cabello et al., 2020; Liu et al., 2015; Simic et al., 1999; Teipel et al., 2005). One interesting study in this sense is the one conducted by Teipel et al. (2005), in which the authors consider the *diminution of signal* in the various areas of the SI, and their correlation with reduced gray matter concentration in different cortical areas.

In AD and other similar diseases, the volume retraction is supposed to be due to the diminution of just the basal ganglion. It must be remembered also that the ganglion extends beyond the limits of the region of the SI. Probably, in the VS, the cholinergic cells are all intrinsic, local circuit neurons, and not projecting neurons to the cortex. On the other hand, they are infrequent, in a ratio of 1/30 with respect to the other cell types (Haber et al., 2011). This means the accumbens and VS should not or almost not atrophy in case of cholinergic cell loss in dementia.

In the case of the EA, the situation could be a little worse: probably a descent of height and volume of the ansa peduncularis in a coronal slice and reduction of the highest portions of the “amygdaloid nucleus” in the zone here analyzed.

The pallidum could be the most affected because the cholinergic cells form part of the different laminae medullaris and also occupy zones immediately below the nucleus.

Furthermore, the volume evaluation of the other components of the zone could help by the characterization and assessment of other psychiatric conditions. As shown, some differences of the tissue signal are so obvious with this sequence that we think it could be a good tool to identify some grey structures in anatomically intricate zones like SI. The similarity of basal ganglion signal with that of the EA is a problem we have tried to resolve to recur in anatomy.

Anatomy is at the base of image interpretation. In this work, we made first a strong anatomical statement based on old and new materials of our series. This allowed us to interpret the IRM tracks of the amygdalofugal way especially the IthP, and the ansa lenticularis without recurrence to tractography. The known relations of the grey substance with fibers, in part of old tradition (Ayala, 1915; Engelhardt, 2013), allowed us to recognize tissue of the basal ganglion associated with tracts, anterior commissure, medullary laminae, and putamen. Never most valuable von Gudden's dictum: *“First anatomy. When physiology first, then never without anatomy.”* We take the license here to change Physiology for Imagenology being sure truthfulness is maintained.

Our MRI study has the remarkable limitation of having been carried out on only one individual. Although we support our views, we consider the interpretation of the images as only provisional and subjected to verification.

Moreover, there remains too much to explore in the region with these or better sequences.

## 6 CONCLUSIONS

This work is exclusively anatomical. We have shown that the fiber structures can be exposed by dissection with magnification without other accessory technical tools. The best approach is with short sessions and meticulous patience. The anatomical stage is essential for the rest of the development of the study.

The structures of this area are highly variable in size intra‐ and inter‐individually.

The APS is a little space where we concentrate essential vessels for the telencephalon, “en passage” or perforating, and neural structures of relevant functional import.

The arrangement of both neural and vascular structures follows a harmonious and topographically describable plan.

The SPACE MRI sequence has proved to be a useful tool to identify fibrous as well as gray substance structures of the zone with acceptable reliability. A previous discriminative anatomical knowledge and a “dissection‐like” attitude are essential to take advantage of its possibilities.

A wider experience with the sequence is imperative to confirm or reject the findings presented here.

## AUTHOR CONTRIBUTIONS

Horacio José Fontana made the dissections and proposed their match with MRI. Juan Mazzucco provided the new material, and stimulated the cerebral anatomical work in ARGUS, he analyzed every imagenological finding, and read and contributed to the final redaction of the article. Sebastián Lescano selected the ES and MRI sequence and participated in the general discussion of the article.

## CONFLICT OF INTEREST STATEMENT

The authors have no conflicts of interest to declare.

## FUNDING INFORMATION

This article was not supported by any funding.

### PEER REVIEW

The peer review history for this article is available at https://publons.com/publon/10.1002/brb3.3029.

## Data Availability

Image data is available.
